# Calcineurin‐Dependent Stress Adaptation Enables Caspofungin Heteroresistance Leading to Stable Resistance in *Candida Glabrata*

**DOI:** 10.1002/advs.76369

**Published:** 2026-06-28

**Authors:** Yanyu Su, Yi Li, Meng Xiao, Dan Guo, David S. Perlin, Erika Shor, Yingchun Xu, Yingxing Li

**Affiliations:** ^1^ Department of Laboratory Medicine State Key Laboratory of Complex Severe and Rare Diseases Peking Union Medical College Hospital Chinese Academy of Medical Sciences and Peking Union Medical College Beijing China; ^2^ Graduate School Chinese Academy of Medical Science and Peking Union Medical College Beijing China; ^3^ National Infrastructures for Translational Medicine Institute of Clinical Medicine Peking Union Medical College Hospital Chinese Academy of Medical Sciences and Peking Union Medical College Beijing China; ^4^ State Key Laboratory of Complex Severe, and Rare Diseases Chinese Academy of Medical Sciences and Peking Union Medical College Beijing China; ^5^ Clinical Biobank Peking Union Medical College Hospital Chinese Academy of Medical Sciences and Peking Union Medical College Beijing China; ^6^ Center For Discovery and Innovation Hackensack Meridian Health 111 Ideation Way Nutley New Jersey USA; ^7^ Biomedical Engineering Facility of National Infrastructures for Translational Medicine Institute of Clinical Medicine Peking Union Medical College Hospital Chinese Academy of Medical Sciences and Peking Union Medical College Beijing China

**Keywords:** calcineurin, *candida glabrata*, caspofungin, heteroresistance

## Abstract

Antifungal heteroresistance has emerged as a clinical challenge across diverse species. In *Candida glabrata*, however, it has rarely been reported. Here, we characterized heteroresistance in a multicenter collection of 156 *C. glabrata* isolates, revealing a 25% prevalence with caspofungin specificity and 37°C dependent phenotypes. Transcriptomic profiling of the heteroresistant subpopulation under escalating drug pressure revealed an adaptive program centered on cell cycle and cell wall integrity, including several members of the calcineurin pathway. Mechanically, we identified that the phenotype was abolished by pharmacological inhibition or Δ*cnb1*, Δ*crz1* genetic deletion, confirming the regulatory role of calcineurin. Crucially, heteroresistance functioned as a reservoir for resistance, with in vitro descendant strains spanning a spectrum of MIC alterations. Mechanistically, we found the heteroresistance phenotype mostly independent of aneuploidy. To decipher the genetic basis, we applied an unbiased machine‐learning framework to genomic data, which not only identified the canonical *FKS2_F659del* mutation but also prioritized novel candidate *PIR2_G149_I167del*, demonstrating its power to uncover drivers of resistance from complex datasets. In summary, our study established a stepwise model of heteroresistance in *C. glabrata*, wherein calcineurin serves as a master regulator that promotes a resistance reservoir, revealing a potential vulnerability that could be exploited to prevent treatment failure.

## Graphical abstract

Caspofungin heteroresistance is prevalent in clinical *Candida glabrata* isolates and depends on calcineurin‐mediated stress adaptation. This transient phenotype serves as a reservoir for resistance evolution, enabling the emergence of stable resistant descendants under prolonged drug pressure. Targeting calcineurin may therefore represent a strategy to prevent treatment failure and resistance development.

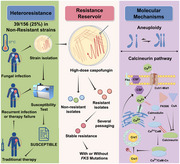

**Abbreviations**
AGE: Agarose Gel Electrophoresis
CFU: Colony Forming Unit
CHIF‐NET: China Hospital Invasive Fungal Surveillance Net
CNV: Copy Number Variation
CsA: Cyclosporin A
GS: Gene Significance
HR: Heteroresistant
MIC: Minimum Inhibitory Concentration
MM: Module Membership
NAT: Nourseothricin
PAP: Population Analysis Profiling
PPI: Protein‐Protein Interactions
RF: Random Forest
RMSE: Root Mean Square Error
ROS: Reactive Oxygen Species
SNP: Single Nucleotide Polyphorism
ST: Sequence Type
WGCNA: Weighted Gene Co‐expression Network Analysis
YPD: Yeast Peptone Dextrose

## 1 Introduction

Invasive fungal infections pose a mounting threat to global public health [1], with *Candida glabrata* (or *Nakaseomyces glabratus*) standing out as a particularly concerning pathogen due to its decreased susceptibility to azoles and its propensity to acquire resistance during antifungal therapy [2, 3]. Echinocandins, such as caspofungin, which target the synthesis of β‐(1,3)‐D‐glucan in the fungal cell wall, have consequently become a cornerstone for treating *C. glabrata* infections [4]. However, the clinical utility of caspofungin is increasingly compromised by the emergence of non‐susceptible strains [5, 6].

Beyond classical, stable resistance conferred by mutations in the *FKS* hotspot regions [6, 7], a more elusive and dynamic phenomenon, heteroresistance, has garnered significant attention. Antifungal heteroresistance is defined as the presence of a resistant subpopulation within a predominantly susceptible isolate, enabling growth at drug concentrations at least eight times of MIC concentration [8, 9]. It is important to distinguish this from the phenomenon of antifungal tolerance. While both may involve survival under drug pressure, fungicidal tolerance typically refers to the ability of the entire population to withstand transient drug exposure without growing [10, 11, 12]. And fungistatic tolerance means the ability to faster overcome the growth inhibition at above‐MIC drug concentrations [12]. Heteroresistance is problematic in *Candida spp*., and in bacteriology, it is considered a potential precursor to full‐fledged, stable resistance [13]. The reversible nature of HR distinguishes it from conventional resistance [11], suggesting a non‐stably heritable, potentially adaptive basis. The molecular mechanisms driving this phenotype, however, remain incompletely elucidated.

In response to cell wall stress induced by drugs like caspofungin, fungi primarily rely on the Cell Wall Integrity and Calcineurin signaling pathways for survival [14, 15, 16]. The latter one, a highly conserved calcium/calmodulin‐dependent signaling cascade [17], is a critical mediator of fungal cell wall stress responses and survival under antifungal pressure [14, 18]. While its role in drug tolerance, persistence, and stable resistance is documented [19, 20, 21, 22], its specific and essential function in the context of caspofungin heteroresistance in *C. glabrata* has not been firmly established.

Furthermore, while heteroresistance represents an immediate survival strategy, its role as a direct reservoir for the evolution of genotypically stable resistance requires direct experimental validation. The genetic determinants that are selected for during this transition, particularly beyond the well‐characterized *FKS* mutations, are poorly defined, leaving a critical gap in our understanding of the resistance evolutionary pathway.

To address these knowledge gaps, we first employed a multi‐center collection of clinical *C. glabrata* isolates to assess caspofungin heteroresistance and then systematically established the calcineurin pathway as its central regulator through pharmacological, genetic, and transcriptomic approaches. We then leveraged the heteroresistant subpopulation in in vitro caspofungin pressure to investigate its role as a precursor to stable resistance. By applying a robust machine learning pipeline to comparative genome sequences of evolved descendant strains (to their parental strains), we aimed to decipher the genetic landscape of resistance development and identify novel drivers beyond the established mechanisms. This integrated study provides a comprehensive view of the resistance continuum in *C. glabrata*, from a calcineurin‐dependent phenotypic adaptation to the acquisition of stabilizing genetic mutations.

## 2 Results

### 2.1 Clinical and Demographic Characteristics of the *C. glabrata* Cohort

**FIGURE 1:**
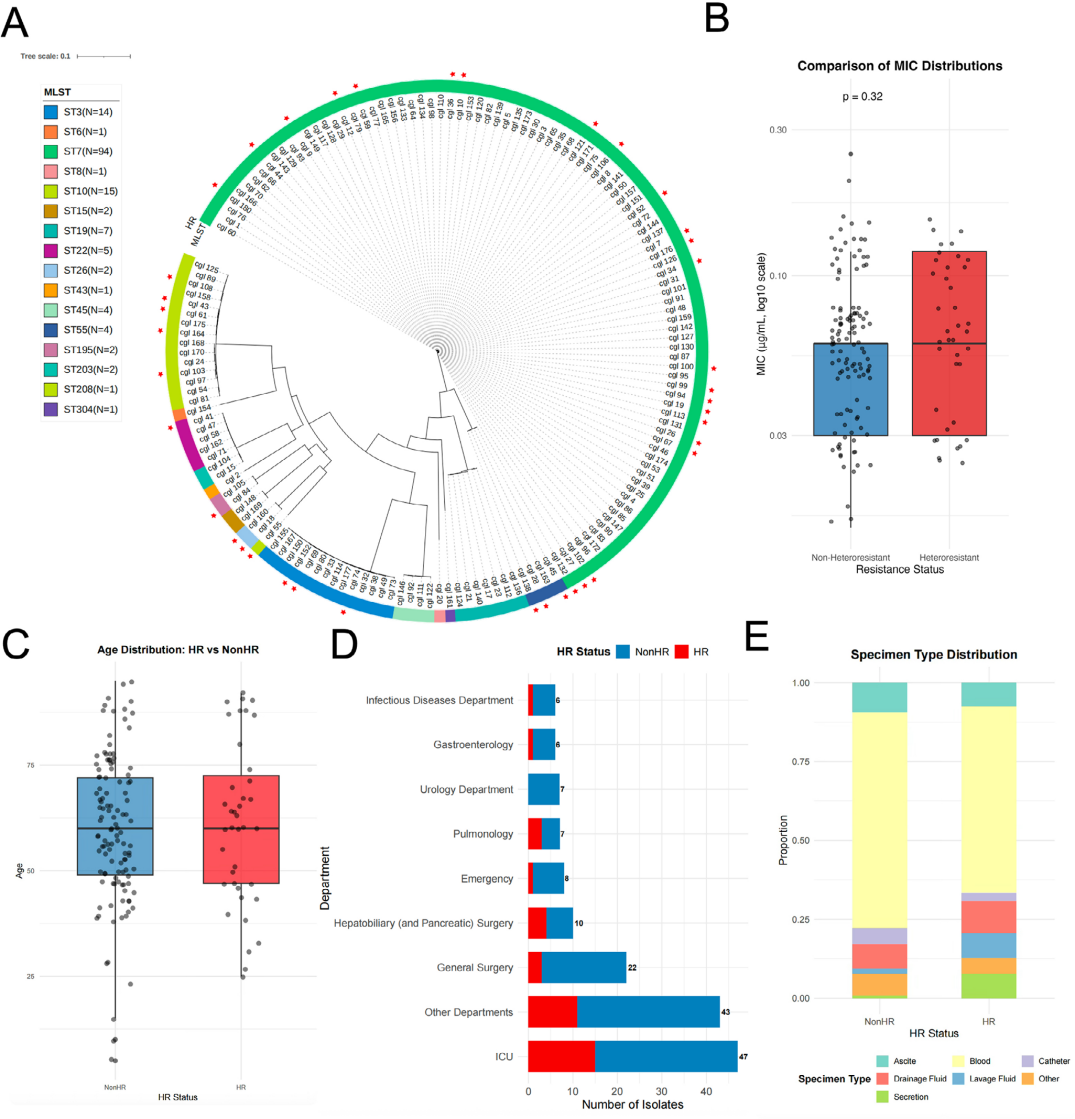
Phylogenic Tree and Epidemical Features of 156 Strains. (A) Phylogenic tree of 156 strains included, consists of 39 HR strains (marked with symbols) and 117 NonHR strains. (B) Caspofungin MIC distribution of HR strains and NonHR strains. (C) Age distribution of patients infected with HR and NonHR strains. (D) Clinical department of patients infected with HR and NonHR strains. (E) Specimen type of HR and NonHR strains.

Our comprehensive epidemiological analysis of 156 invasive infection *C. glabrata* isolates from CHIF‐NET program 2014–2019 (Table S1), revealing important insights into the distribution and characteristics of heteroresistant strains within this collection (Figure 1A). All strains with ≥ 2 visible colonies under at least eight times of the MIC for the most sensitive cells is considered heteroresistant. According to this criterion, the cohort comprised 39 HR isolates (25.0%) and 117 NonHR isolates (75.0%), providing a substantial basis for comparative analysis. Notably, the distribution of caspofungin MIC values did not differ significantly between HR and NonHR isolates (*p* = 0.322), with both groups exhibiting identical median MICs of 0.06 mg/L (Figure 1B). Other statistical evaluation using appropriate tests demonstrated no significant associations between heteroresistance status and key demographic or clinical variables. Patient age distribution was comparable between HR and NonHR groups (Figure 1C), with mean ages of 61.2 and 59.9 years, respectively (*p* = 0.705). Similarly, gender distribution showed no significant difference, with females comprising 43.6% in both HR and NonHR populations (*p* = 1.000).

Among these invasive infection isolates, the top three specimen sources were blood (103/156, 66.03%), ascites (14/156, 8.97%), and drainage fluid (13/156, 8.33%). The departmental distribution analysis revealed that ICU settings accounted for 43.6% of HR isolates compared to 29.9% of NonHR isolates, though this difference did not reach statistical significance (*p* = 0.170, Figure 1D), probably due to the relatively small size of our cohort. Specimen type analysis, focusing on the six most prevalent sample sources (Blood, Drainage Fluid, Ascites, Lavage Fluid, Catheter, and Secretion), revealed no significant association with heteroresistance status (*p* = 0.130, Figure 1E). Complete statistical analyses for all specimen types are provided in Table S2. Genetic characterization of the collection identified 15 distinct sequence types by multilocus sequence typing, with ST7 predominates (94/156, 60.26%). Whole‐genome SNP phylogeny confirmed that isolates broadly clustered within their designated sequence types, validating the MLST‐based classification (Figure 1A). Despite this genetic diversity, statistical analysis revealed no significant association between sequence type and heteroresistance status (*p* = 0.077), suggesting that heteroresistance occurs across diverse genetic backgrounds.

This comprehensive epidemiological profiling demonstrates that the heteroresistance phenotype in *C. glabrata* is not strongly associated with specific patient demographics, clinical settings, or major genetic lineages within our collection. The widespread distribution of heteroresistance across diverse sequence types suggests that this phenotype represents a convergent evolutionary adaptation rather than being restricted to specific clonal lineages. The absence of strong epidemiological predictors underscores the complexity of heteroresistance development and highlights the need for mechanistic investigations to identify the underlying molecular determinants driving this phenotype across diverse genetic backgrounds.

### 2.2 Dynamic and Conditional Features of Caspofungin Heteroresistance

**FIGURE 2:**
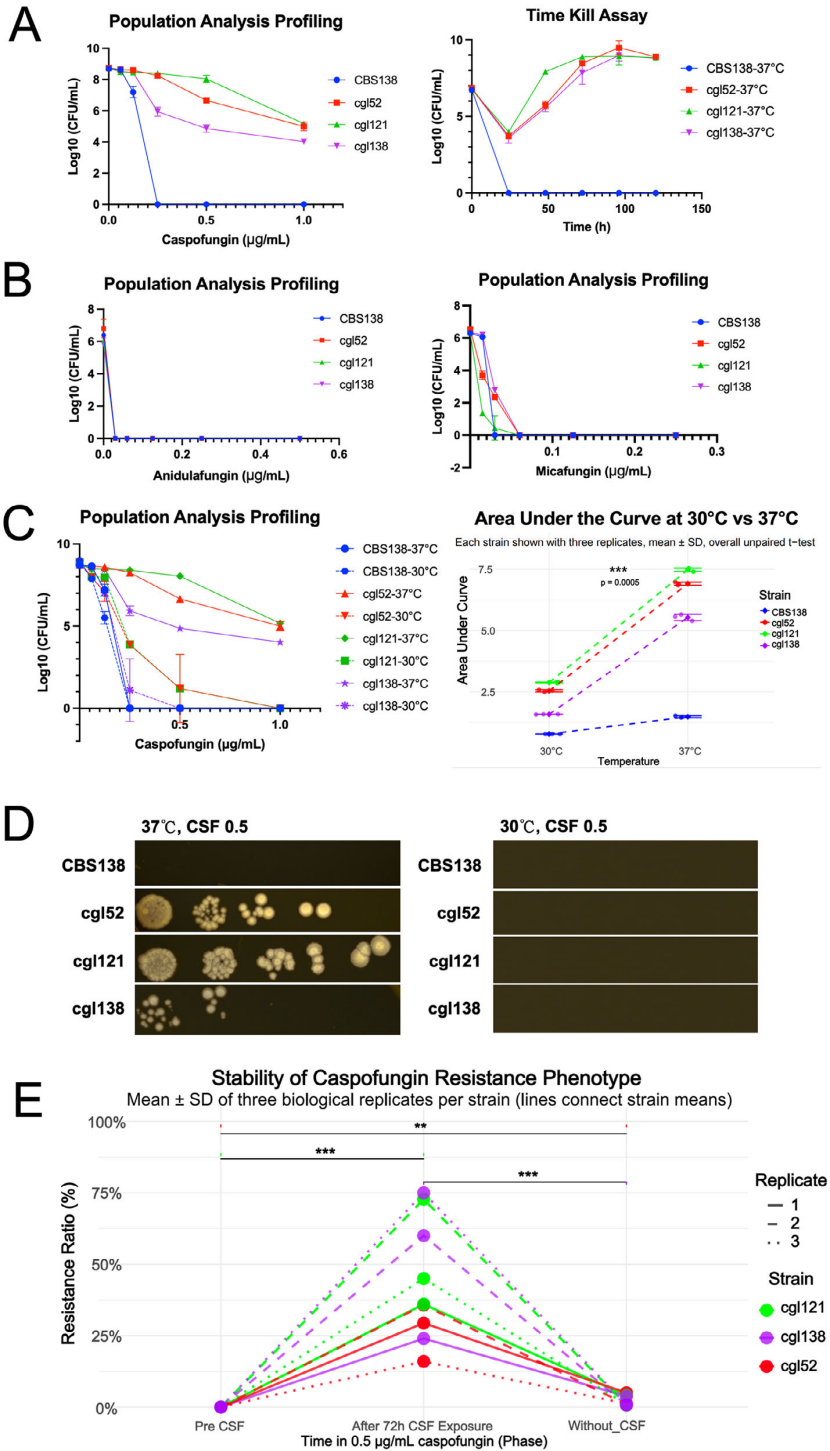
Phenotypic and Hereditary Features of Heteroresistant Strains. (A) HR isolates exhibited extended growth on agar plates with serial concentration of caspofungin and a distinctive “V‐shaped” or rebound phenotype in time‐kill assays, all under 37°C. (B) Caspofungin HR isolates exhibited no extended growth on agar plates containing anidulafungin or micafungin. (C) PAP curves of cgl52, cgl121, cgl138, and statistical analysis of AUCs among all 39 HR strains under 30°C and 37°C. (D) PAP spot assays of representative isolates on YPD containing 0.5 µg/mL CSF under different temperatures, with no visible colonies under 30°C. (E) Hereditary stability of HR strains before, during, and after high‐dose caspofungin pressure, indicating the reversibility of HR phenotype. ^*^ *p* < 0.05; ^**^ *p* < 0.01; ^***^ *p* < 0.001; ^****^ *p* < 0.0001.

To dynamically characterize the response of *C. glabrata* to caspofungin and separate this phenomenon from tolerance or persistence, we performed time‐kill assays on a representative subset of isolates that consists of highly heteroresistant isolate cgl121, moderately heteroresistant isolate cgl52, and mildly heteroresistant isolate cgl138. This analysis revealed a striking divergence in killing kinetics between HR and NonHR strains (Figure 2A). Against reference strain CBS138, 0.5 µg/mL caspofungin exerted a sustained effect, with viable counts remaining below the baseline soon after the drug was added. In stark contrast, HR isolates exhibited a distinctive “V‐shaped”, rebound phenotype. After an initial decline in CFUs that reached its nadir at 24 h, a robust regrowth phase ensued, with viable counts returning to, and in some cases exceeding, the starting inoculum by 120 h. We at the same time conducted a drug‐free control, confirming that this was totally a result of a resistant subpopulation under a high dose of caspofungin (Figure S1A). This reproducible rebound trajectory provides direct evidence that the HR subpopulation is not merely tolerant but can proliferate under ongoing drug pressure [8], underscoring the dynamic and adaptive nature of heteroresistance.

A key question was whether heteroresistance represents a general response to the echinocandin class of antifungals or is drug‐specific. At our first screening, we performed PAP assays on the three commonly used echinocandins clinically, anidulafungin, micafungin, and caspofungin, using each of their non‐resistant strains according to our antimicrobial susceptibility test results (caspofungin MIC < 0.5, micafungin MIC < 0.25, anidulafungin MIC < 0.5, Table S3). Notably, all strains showed no evidence of heteroresistance when challenged with anidulafungin or micafungin, even those exhibited a robust HR phenotype to caspofungin at 37°C (PAP curves of representative strains are shown in Figure 2B, PAP spot assays in Figure S1B, and time‐kill assays in Figure S1C). Though there are certain subpopulations that can survive under micafungin concentration above MIC, they still fail to form visible colonies at eight times of its own MIC (Figure S1B). The phenotype of these strains displayed a classic susceptible profile, with a sharp decline in CFUs at concentrations near the MIC, and no rebound growth during the killing process (Figure S1C). This clear distinction indicates that the ability to form a resistant subpopulation is a drug‐specific characteristic unique to caspofungin within the echinocandin class in *C. glabrata* and is not a general mechanism of survival against cell wall synthesis inhibition.

We next investigated whether physiological factors, particularly temperature, modulate the HR phenotype in *C. glabrata*. While initial PAP screening at the host‐relevant temperature of 37°C revealed a robust HR phenotype, parallel assays at 30°C (often associated with environmental adaptation) demonstrated a profound temperature dependence (Figure 2C). For all 37°C‐screened HR strains, the phenotype was abolished at 30°C: they failed to form resistant subpopulations on agar plates with eight times of its populational MIC concentration of caspofungin (spot assays of representative strains are shown in Figure 2D and Figure S2A,B), erasing this dynamic hallmark of heteroresistance. Quantitative PAP analysis confirmed a significant reduction in the surviving subpopulation at elevated drug concentrations for all strains at 30°C (Figure 2C,D). Importantly, the baseline MICs remained unchanged across temperatures (Table S4), confirming that temperature specifically modulates the heteroresistance trait without affecting population‐wide susceptibility. These findings establish caspofungin heteroresistance in *C. glabrata* as a strongly temperature‐dependent phenomenon, predominantly expressed under host physiological conditions.

Having established its phenotype, we sought to determine the genetic stability of heteroresistance. We explored the resistance ratio before, during, and after caspofungin pressure. Prior to caspofungin exposure, the proportion of cells capable of growing on 0.5 µg/mL caspofungin was minimal across the six tested parental strains (< 0.1%), confirming the rare nature of the resistant subpopulation (Figure 2E). Following 48‐h exposure to 0.5 µg/mL caspofungin in liquid culture, this resistant subpopulation was dramatically enriched, with the resistance ratio increasing significantly to a mean of 43.76% ± 21.13% (range: 16.00‐75.00%, median: 36.00%; *p* = 0.0003 vs. pre‐exposure). Crucially, when these enriched populations were subsequently passaged in drug‐free medium for 72 h, the proportion of resistant cells decreased significantly to a mean of 1.99% ± 1.74% (range: 0.65%–5.00%, median: 0.89%; *p* = 0.0005 vs. during drug exposure, *p* = 0.0093 vs. pre drug exposure), indicating that the resistance phenotype was largely plastic and reversible in the absence of continuous selective pressure. But compared to the initial population, after drug exposure, the resistance subpopulation is significantly up regulated (*p* = 0.0093, pre‐exposure vs. after exposure). This positions this adaptable subpopulation as a potential reservoir that can be rapidly mobilized upon drug challenge while maintaining the potential for phenotypic reversion in the absence of selective pressure.

### 2.3 The Heteroresistant Subpopulation Exhibits a Distinct Transcriptional Signature

**FIGURE 3:**
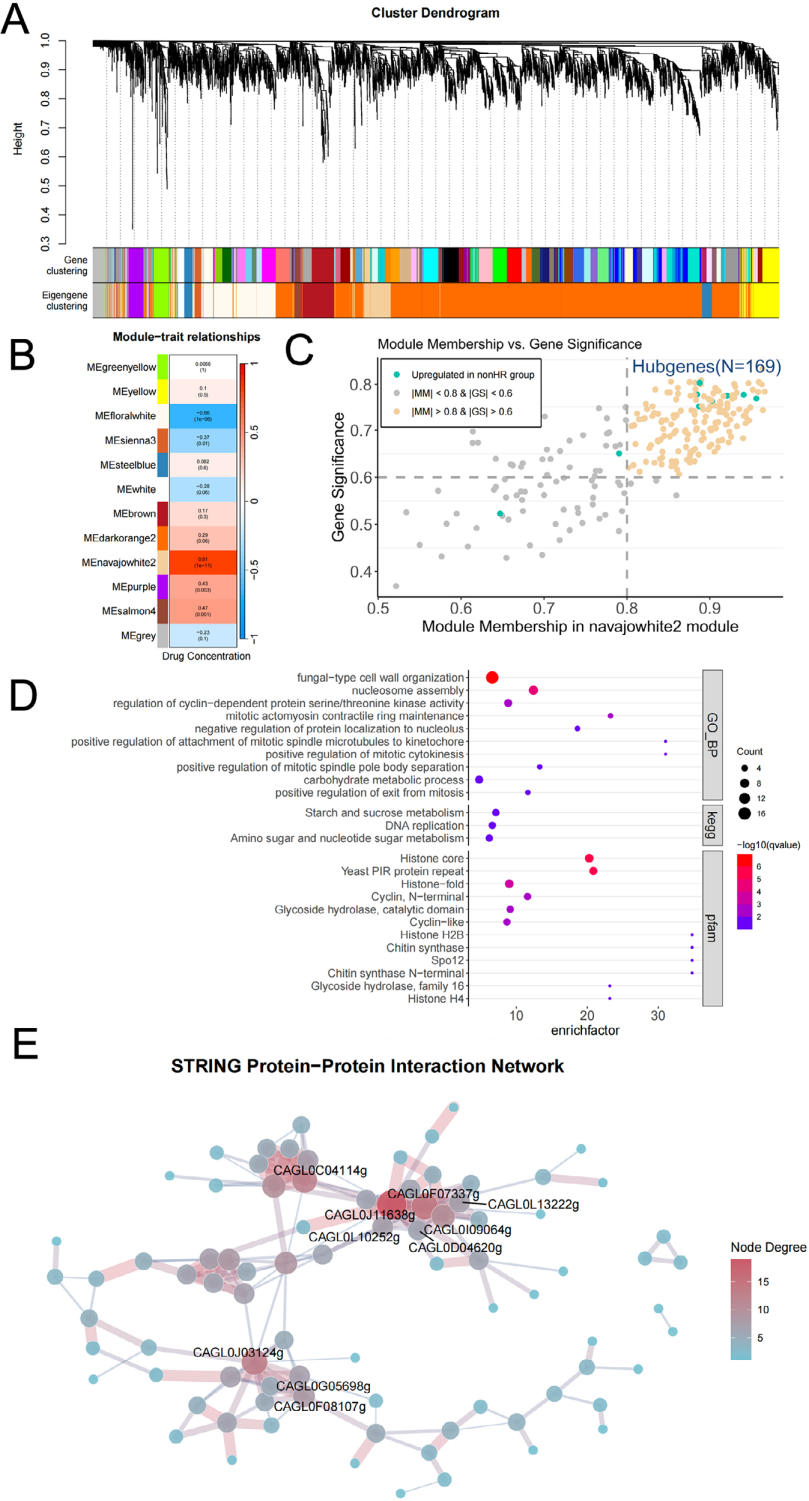
Transcriptional Network Analysis Reveals Core Functional Modules in Caspofungin Heteroresistance. (A) Overview of the WGCNA performed on transcriptomes from heteroresistant *C. glabrata* isolates under escalating caspofungin pressure. (B) Module–trait relationships showing the correlation between module eigengenes and drug concentration. One key module (highlighted) exhibited a strong positive correlation with increasing caspofungin concentration (Pearson's r > 0.8, *p* <1e^−10^). (C) Illustration of the key module comprised 248 genes, of which 169 were defined as hub genes based on high module membership (MM > 0.8) and high gene significance (GS > 0.6). Genes that were upregulated in NonHR strains under drug pressure were colored in green. (D) Functional enrichment analysis of the 169 hub genes. The top significantly enriched terms are shown, highlighting cell cycle progression as one of the core adaptive processes. (E) PPI network and visualization of hub genes, with *CAGL0J11638g* emerged as the most central node.

To define the transcriptional underpinnings of survival under caspofungin pressure, we performed RNA sequencing on five HR isolates (0, 0.125, and 1 µg/mL) exposed to escalating drug concentrations. WGCNA of the resulting transcriptomes identified one key module whose eigengene expression exhibited a strong positive correlation with increasing caspofungin concentration (Pearson's r > 0.8, *p* < 1e^−10^; Figure 3A,B). This module comprised 248 genes, representing a core transcriptional response to drug stress (Figure 3C).

To pinpoint the most biologically relevant drivers within this module, we calculated Module Membership and Gene Significance, correlation with the module eigengene, and drug concentration, respectively. Excluding those up‐regulated in NonHR strains under low caspofungin concentration, 162 genes with high connectivity (MM > 0.8 or < −0.8) and high trait relevance (GS > 0.6 or < −0.6) were identified as hub genes (Table S5). Functional enrichment analysis revealed that these hub genes were overwhelmingly enriched for processes related to cell wall integrity and cell cycle regulation (Figure 3D). This indicated a concerted adaptive strategy: fortifying the primary drug target site while sustaining proliferative capacity.

To understand the functional relationships among these hub genes, we constructed a PPI network using the STRING database (https://cn.string‐db.org/). Using the cytoHubba plugin, we ranked genes by their network connectivity and identified the top 10 hub genes within the PPI network (Figure 3E). Among these, *CAGL0J11638g* (ranked first, the ortholog of *S. cerevisiae CDC5*), which mediates centromeric DNA binding and phosphoprotein binding, emerged as the most central node. Given this, we attempted to generate gene deletion mutants to assess its functional role in heteroresistance. However, repeated genetic knockout attempts were unsuccessful across multiple genetic backgrounds. This failure to obtain viable null mutants is consistent with phenotypic annotations in the Candida Genome Database (http://www.candidagenome.org/), which classify this gene as well as *CAGL0F07337g* (rank second and has anaphase‐promoting complex binding, ubiquitin ligase activator activity; the ortholog of *S. cerevisiae CDC20*) and *CAGL0D04620g* (ranked third and has cyclin‐dependent protein serine/threonine kinase activator and regulator activity; the ortholog of *S. cerevisiae CLB1*) as “inviable” [23], indicating they are likely essential genes for *C. glabrata*. This essentiality underscores the hub genes’ critical biological function and suggests that their upregulation under drug pressure may represent a non‐dispensable component of the heteroresistance survival program.

Notably, within the up‐regulated 248‐gene, we identified several genes known to be regulated by the calcineurin/Crz1 pathway, including *CHS3* (encoding chitin synthase III), *UTR2*, *ALG5*, etc., which showed significant upregulation with increasing caspofungin concentration [24, 25]. *CHS3* expression has been shown to depend on Crz1 under Ca^2^^+^ stress, and its disruption leads to cell wall integrity defects [24]. In *Candida albicans*, *UTR2* is a direct target of the calcineurin/Crz1 pathway and is induced in response to cell wall stress [25]. Moreover, independent genetic screens have shown that deficiencies in endoplasmic reticulum N‐glycosylation genes such as *ALG5* confer hypersensitivity to calcineurin inhibitors, indicating that these processes engage calcineurin‐dependent stress adaptation [26]. This transcriptional evidence suggested that the calcineurin pathway might play a role in orchestrating the HR‐associated adaptive program. To directly test this hypothesis, we proceeded to genetically dissect the calcineurin pathway through drug intervention and gene deletions.

### 2.4 Caspofungin Heteroresistance is Dependent on Calcineurin Pathway

**FIGURE 4:**
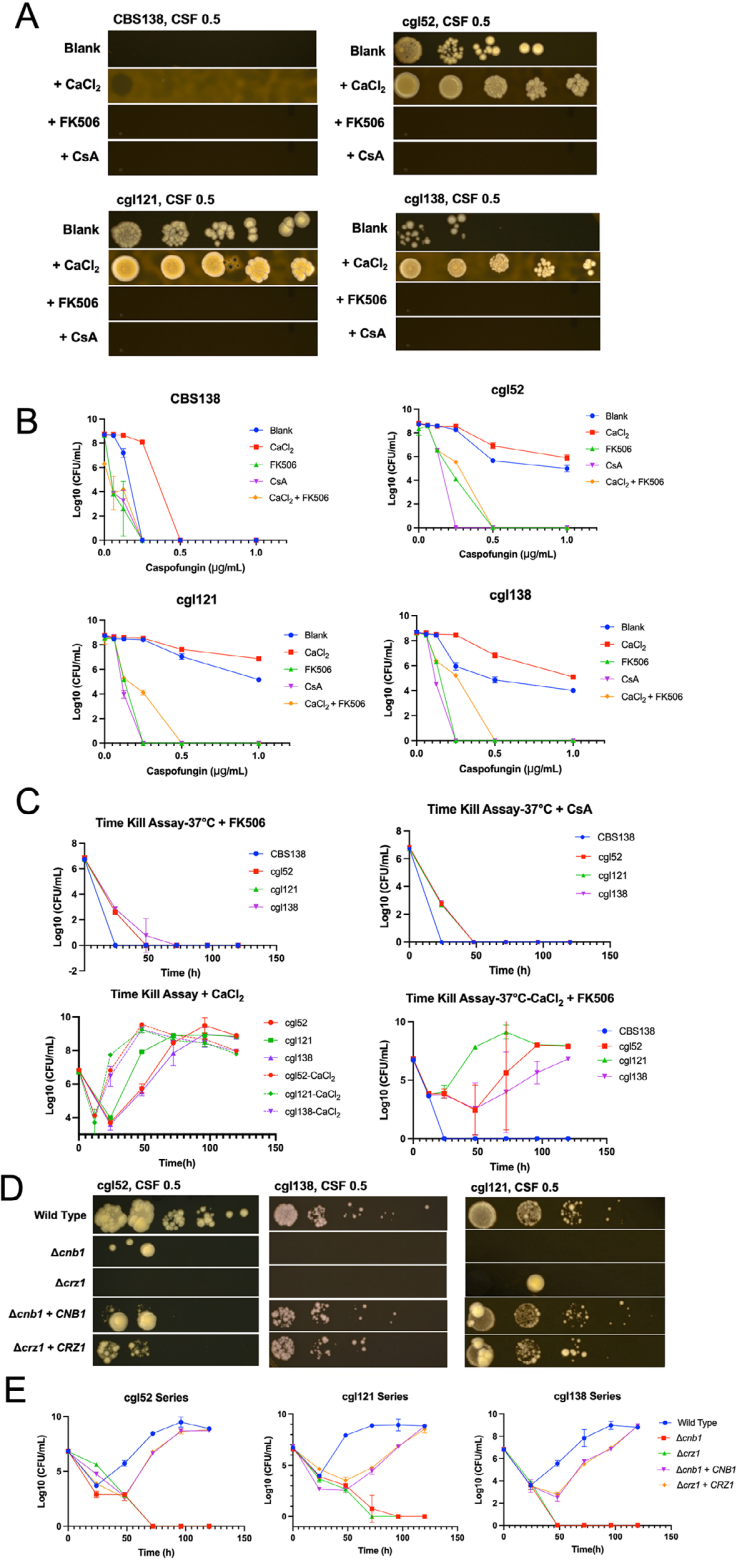
Caspofungin Heteroresistance is Enhanced by Calcium Supplementation and Abolished by FK506 Inhibition. (A) PAP spot assays of representative HR isolates on YPD agar containing 0.25 µg/mL caspofungin, either alone or supplemented with CaCl_2_ or FK506. (B) Quantitative PAP curves of isolates under four conditions: CSF alone, CSF + CaCl_2_, CSF + FK506, and CSF + CaCl_2_ + FK506. (C) Time‐kill curves of HR strains under 0.5 µg/mL caspofungin with CaCl_2_, FK506, CsA, or combined intervention. (D) PAP spot assays on YPD agar containing 0.5 µg/mL caspofungin for wild‐type, null mutants (Δ*crz1*, Δ*cnb1*), and complemented strains of three representative clinical isolates (cgl52, cgl121, cgl138). (E) Time‐kill curves of wild‐type, null mutants (Δ*crz1*, Δ*cnb1*), and complemented strains of three representative clinical isolates (cgl52, cgl121, cgl138).

Having established the phenotypic features of heteroresistance, we next sought to identify whether the calcineurin pathway, a central integrator of calcium signaling and cell wall integrity [18, 27, 28], modulates heteroresistance. Strikingly, pharmacological inhibition of calcineurin with FK506 completely abolished the phenotype (Figure 4A,B). This observation pointed to calcineurin as a candidate regulator of caspofungin heteroresistance in *C. glabrata*, as FK506, upon binding its intracellular receptor FKBP12, specifically inhibits calcineurin phosphatase activity—a mechanism conserved from yeast to humans and well‐established in fungal pathogens [29].

To consolidate this finding and exclude potential off‐target effects of FK506, we tested cyclosporin A (CsA), which inhibits calcineurin through a distinct immunophilin (cyclophilin A) [30]. CsA phenocopied the effect of FK506, completely abolishing the heteroresistant subpopulation in PAP assays (Figure 4A,B). The essential role of calcineurin was further underscored by time‐kill kinetics: while the parental HR strains exhibited the characteristic rebound growth under caspofungin pressure, both FK506 and CsA eliminated this rebound, rendering the killing curve indistinguishable from that of a susceptible strain (Figure 4C). These results confirm that the calcineurin phosphatase itself, rather than any upstream component unique to the FK506‐FKBP12 complex, is the critical node governing caspofungin heteroresistance in *C. glabrata*.

We next asked whether activation of the calcineurin pathway, instead of its inhibition, could reciprocally potentiate heteroresistance. As shown by PAP spot assays, supplementation with 200 mm CaCl_2_ markedly expanded the resistant subpopulation even in CBS138, enabling robust growth at caspofungin concentrations that otherwise suppressed the entire population (Figure 4A,B). This rescuing effect was also evident in time‐kill kinetics: in the presence of 100 mm CaCl_2_, the rebound growth characteristic of HR strains was accelerated, with the nadir of viable counts shifting from 24 h to 8 h (Figure 4C). These observations demonstrate that sufficient calcium signaling, likely through activation of the calcineurin cascade, potentiates the heteroresistant phenotype, further supporting calcineurin as a central regulator of this adaptive trait.

A critical question was whether this calcium‐mediated rescue represented a transition to stable, genetic resistance. To distinguish this, we isolated individual colonies that grew on high‐dose caspofungin plates (0.5 µg/mL) supplemented with CaCl_2_, designated as “rescued” colonies, and subjected them to standard broth microdilution MIC testing in the absence of CaCl_2_. The MICs for these rescued clones were unchanged from the baseline MIC of the parent HR strain or did not meet the criteria for resistance (Table 1). This confirmed that calcium rescue sustains a reversible, phenotype‐dependent survival mechanism, rather than selecting for or inducing stable resistance. This interpretation aligns with the established model wherein increased intracellular calcium activates calmodulin and subsequently the calcineurin phosphatase, a pathway increasingly recognized for its role in regulating antifungal tolerance and persistence [20].

**TABLE 1:** Caspofungin MICs of original and calcium‐rescued strains.

Strains/MIC [µg/mL]	CBS138	cgl52	cgl138	cgl121
Parent Strain	0.06	0.03	0.12	0.12
Rescued Colony 1	0.06	0.03	0.06	0.25
Rescued Colony 2	0.06	0.03	0.06	0.12
Rescued Colony 3	0.06	0.03	0.06	0.12

To comprehensively test this model of a calcineurin‐dependent route, we also employed a combinatorial intervention, assessing heteroresistance on caspofungin gradient plates supplemented with both FK506 and CaCl_2_. Interestingly, exogenous calcium was able to partly rescue the heteroresistant subpopulation even in the continuous presence of the calcineurin inhibitor FK506 (Figure 4B). The mild growth observed under these co‐treatment conditions suggests that elevated extracellular calcium can partially bypass calcineurin dependence, either through a parallel, calcium‐activated pathway or by enhancing populational stress resilience independently of calcineurin signaling. Distinguishing between these mechanisms will require further investigation.

To genetically explore how the calcineurin pathway interacts with caspofungin stress, we abolished its biological function of it through gene deletion, of which *CNB1* and *CRZ1* were chosen [31]. Δ*Cnb1* and Δ*crz1* null mutants were constructed in reference strain CBS138 and HR strains cgl52, cgl121, and cgl138. Deletion of these genes did not cause significant changes in caspofungin MICs (Table 2), which transiently excluded the effect of whole‐populational susceptibility. Genetic dissection of the calcineurin pathway through targeted deletion of *CNB1* and *CRZ1* revealed a striking calcineurin‐dependent homogeneity in their contributions to heteroresistance, as Δ*cnb1* and Δ*crz1* null mutants nearly fail to form visible colonies under the concentration of 0.5 µg/mL (caspofungin) but plasmid complementation rescues the phenotype, though may not be the exact same with respective wild type strains (Figure 4D). Consistent with the PAP assays, time‐kill curves of the Δ*cnb1* and Δ*crz1* mutants showed no rebound growth, confirming the eradication of the heteroresistant subpopulation (Figure 4E). In contrast, the complemented strains exhibited low colony counts at 24 and 48 h but demonstrated a discernible recovery, thereafter, resulting in a right‐shifted, attenuated “V‐shaped” regrowth curve reminiscent of the parental HR phenotype (Figure 4E).

**TABLE 2:** Caspofungin MICs of mutant strains and complemented strains.

Strains/MIC [µg/mL]	CBS138	cgl52	cgl138	cgl121
Wild Type Strain	0.06	0.03	0.12	0.12
Δ*cnb1*	0.06	0.03	0.12	0.25
Δ*crz1*	0.06	0.03	0.12	0.12
Δ*cnb1* + *CNB1* complementation	0.06	0.03	0.12	0.12
Δ*crz1* + *CRZ1* complementation	0.06	0.03	0.06	0.12

### 2.5 Heteroresistant Subclone as a Reservoir for Caspofungin Resistance

In our previous research, an initially susceptible HR strain, cgl180, evolved to resistance during the infection course [32]. In the current study, having established that the calcineurin pathway underpins a transient, high‐frequency HR phenotype in clinical isolates, we sought to determine its clinical consequence: does this phenotypically resistant subpopulation serve as a precursor for the development of genotypically stable resistance?

**FIGURE 5:**
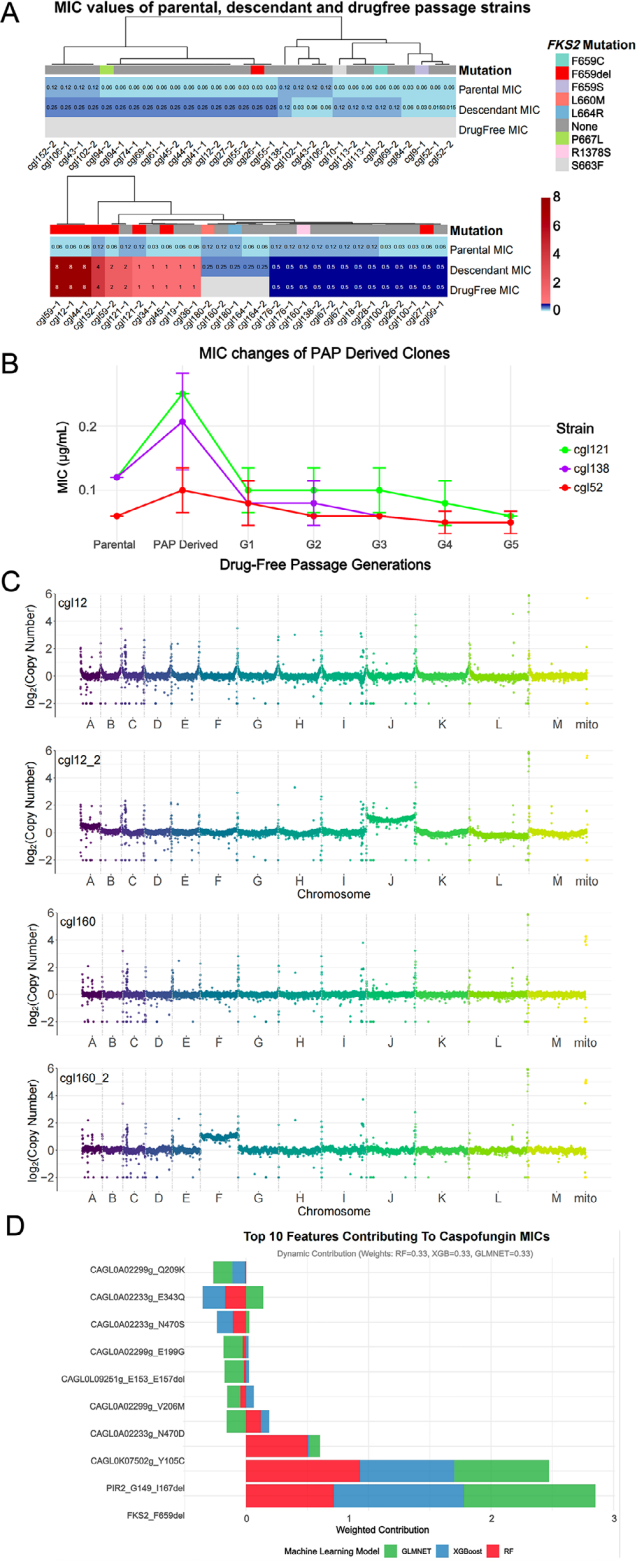
Comparative Analysis and Top Features Screened by Multi‐Generative Machine Learning Models. (A) Comparison of parental, descendant, and drug‐free passage caspofungin MIC trend. (B) MIC changes of PAP‐derived clones under drug‐free conditions. (C) Chromosome copy number variation of cgl12‐2 compared to cgl12, with chrJ amplified, and chromosome copy number variation of cgl160‐2 compared to cgl160, with chrF amplified. (D) Top 10 features screened by generative model, with RF, XGBoost, GLMNET each weighting 33.33%. ^*^ *p* < 0.05; ^**^ *p* < 0.01; ^***^ *p* < 0.001; ^****^ *p* < 0.0001.

To determine whether the subpopulation that survived high‐dose caspofungin pressure (1 µg/mL) on solid medium had already acquired genetically stable resistance, we first isolated 59 clones from 35 parental HR strains that formed colonies on YPD agar containing 1 µg/mL caspofungin. These clones were then expanded in liquid YPD medium containing 0.5 µg/mL caspofungin to obtain sufficient biomass for subsequent analyses. For all descendants, their caspofungin susceptibility level spanned a continuous spectrum (Figure 5A, Table S6). A significant subset was with profoundly elevated MICs, including superior‐resistant phenotypes (MIC > 8 µg/mL, 3/59, 5.08%), like cgl12‐1, cgl59‐1, etc., and resistant phenotypes (0.5 ≤ MIC < 8, 21/59, 35.59%). Others attained moderate resistance or just an increase in MIC compared to their parental ancestors while remaining below the formal resistance breakpoint (MIC < 0.5, 28/59, 47.46%). Importantly, this progression was not universal; a minority of clones retained their original susceptibility or even increased their susceptibility (7/59, 11.86%), like cgl9‐1 (from 0.06 to 0.03), cgl52‐1 (from 0.06 to 0.015), cgl52‐2 (from 0.06 to 0.03), etc. Notably, all 24 descendant strains with MIC ≥ 0.5 µg/mL maintained their elevated MICs after five serial drug‐free passages (Table S6), indicating that they had acquired genetically stable resistance during the two‐step selection process. The emergence of this full spectrum of resistance levels, from superior‐resistant to increased susceptibility, from a uniformly HR starting population provides direct experimental evidence that the heteroresistant subpopulation acts as a phenotypic reservoir. It is from this reservoir that stable resistance genotypes are selectively enriched under drug pressure, thereby bridging a reversible phenotypic adaptation to the emergence of clinical resistance.

However, because the MICs of the primary clones immediately after isolation from 1 µg/mL plates were not determined, it remained unclear whether the stable resistance observed in these descendants arose directly on the high‐dose plates or only after subsequent liquid culture in 0.5 µg/mL caspofungin. To address this question, we performed a controlled experiment using three representative HR strains (cgl52, cgl121, and cgl138). Each strain was subjected to PAP on YPD agar containing 1 µg/mL caspofungin, and three independent colonies per strain were picked directly from the plates (PAP‐derived clones). These clones were immediately tested for caspofungin MIC and then passaged five times on drug‐free YPD agar to assess stability.

Strikingly, none of the PAP‐derived clones exhibited MICs reaching 1 µg/mL; their MICs ranged from 0.06 to 0.25 µg/mL (Figure 5B). Moreover, after five passages in drug‐free medium, all clones with MIC increased compared to parental strains showed a decrease in MIC by at least one two‐fold dilution, indicating that the elevated MICs observed immediately after isolation were not genetically stable. Thus, although these cells could survive transient exposure to 1 µg/mL caspofungin on solid medium, their reduced susceptibility was largely attributable to reversible, adaptive mechanisms rather than fixed genetic alterations. Thus, survival on 1 µg/mL caspofungin plates is primarily mediated by phenotypic adaptation, whereas genetically stable resistance emerges only upon sustained drug pressure in liquid culture containing 0.5 µg/mL caspofungin. This stepwise process, from transient adaptation to stable genetic resistance, underscores the critical role of heteroresistance as a reservoir for the evolution of clinical echinocandin resistance.

### 2.6 Caspofungin Heteroresistance is Largely Independent of Aneuploidy in *C. glabrata*

Among all isolated stably resistant descendants, a considerable proportion (16/24, 66.7%) did not carry *FKS2* mutations that have been reported to confer reduced susceptibility. This observation prompted us to further examine whether additional determinants, independent of or contributing to *FKS2*‐mediated caspofungin resistance, were involved. We performed whole‐genome sequencing on the above‐mentioned descendant strains, comparing them to the original strains. Given that aneuploidy has been implicated in antifungal resistance and stress adaptation in related fungal pathogens [33, 34, 35], we first systematically assessed chromosome copy number variations (CNVs) across our isolate collection.

Our analysis demonstrates that the caspofungin HR phenotype in *C. glabrata* is not universally dependent on gross aneuploidy. Most descendants maintained a stable euploid karyotype (43/59, 72.88%), underscoring that alternative, likely signaling‐based mechanisms suffice to confer the phenotype. This finding positions caspofungin heteroresistance in *C. glabrata* in sharp contrast to the mechanism described for *C. albicans* and *C. neoformans* heteroresistance, where aneuploidy is a common driver [36, 37].

However, in a subset of cases, we identified specific aneuploidy events, such as amplification of Chromosome G, F, J, etc. (16/59, 27.12%; Figure 5C; Tables S7 and S8), which were present in the descendant subclone but absent in the matched parental population. A recurrent pattern of chrJ amplification was observed in three strains cgl12‐2, cgl26‐2, and cgl138‐1 (Figure 5C), indicating that clonal aneuploidy may serve as one of several potential genomic strategies to generate a resistant subpopulation within an otherwise susceptible isolate, whether pre‐existing or induced. However, aneuploidies of other chromosomes appeared stochastic with no consistent pattern, suggesting it is a strain‐specific strategy rather than a conserved mechanism. Apart from this, 12 out of 59 (20.34%) descendant strains carry proved clinically significant *FKS2* point mutations, including *F659del*, *F659S*, *F659C*, and *S663F* [38, 39, 40]. Strains harboring the *F659del* mutation exhibited MIC elevations ranging from 8‑ to 256‑fold. The *F659S*, *F659C*, and *S663F* variants were each identified in only one strain, associated with a reduction in MIC (to half of the parental level), a twofold increase, and a fourfold increase, respectively. Other *FKS2* mutations observed—*R1378S*, *L664R*, *L660M*, *R1378H*, and *P667L*—have not been established to correlate with echinocandin susceptibility. These suggest that while a stable euploid genome is permissive for heteroresistance, which is largely mediated by the calcineurin pathway, preexisting aneuploidy in a minority of isolates represents a parallel, genetic route to achieve the same transiently resistant phenotype.

### 2.7 *PIR2_G149_I167del* as a Novel Candidate Determinant of Caspofungin MIC

To systematically decipher the genetic landscape underlying caspofungin resistance, we employed a robust machine learning framework integrating a multi‐stage feature selection pipeline with an ensemble of predictive models. This approach was designed to handle the high‐dimensionality of our whole‐genome sequencing data and robustly identify mutations with the strongest association to the resistance phenotype (STATUS). The final ensemble model, which aggregated predictions from Random Forest, XGBoost, and Elastic Net, demonstrated proficient performance in predicting the continuous MIC fold‐change, achieving an R^2^ of 0.846 and a root mean square error (RMSE) of 0.885 on the held‐out test set, confirming its utility for this genetic screening task.

A critical validation of our machine learning pipeline's efficacy was its successful recapitulation of the first‐ranked gene *FKS2* (*CAGL0K04037g_F659del*), the gene encoding the catalytic subunit of the echinocandin target enzyme β‐(1,3)‐D‐glucan synthase (Figure 5D). This specific mutation has also been reported for its contribution to elevated echinocandin MICs [38, 41]. This canonical resistance gene was ranked as the first important feature by our model. Independent statistical validation through continuous enrichment analysis revealed that its *F659del* mutation demonstrated a strong positive correlation (r = 0.573, *p* = 0.0009) and the largest absolute effect size (3.316) across all features in the dataset (Table S9). The high‐ranking identification of this established resistance mechanism underscores the predictive power and biological relevance of our computational strategy.

Apart from *FKS2*, our analysis yielded a highly prioritized list of genetic determinants (Table S9), with a previously uncharacterized mutation in the cell wall‐related gene *PIR2* (*CAGL0I06182g*, *PIR2_G149_I167del*) ranking just after *FKS2* (Figure 5D). The *PIR2_G149_I167del* mutation possessed a strong positive correlation with STATUS (r = 0.542, *p* = 0.0014) and conferred a “Very Large” effect size of 2.485 (Table S9). And it is up regulated under escalating doses of caspofungin, as analyzed in our transcriptomic sequencing. This indicates that strains harboring this specific mutation exhibited a markedly elevated MIC ratio compared to their wild‐type counterparts.

To functionally validate the causal role of the top‐ranked mutation identified by our machine learning model, we performed precise genetic manipulations in selected strains. Using homologous recombination, we generated *PIR2* knockout mutants (Δ*pir2*) in three descendant strains harboring the *PIR2_G149_I167del* mutation (cgl19‐1, cgl36‐1, cgl100‐1) and two control strains lacking this mutation (the reference strain CBS138 and clinical isolate cgl121).

The impact on caspofungin susceptibility is mild but specific, and no alteration in micafungin or anidulafungin is detected (Table 3). Interestingly, all strains with a mutated *PIR2* gene showed a one‐fold caspofungin MIC decrease in Δ*pir2* strains, with cgl121 and CBS138 showed no MIC change. To unequivocally link the resistance phenotype to the specific *PIR2* mutation, we performed plasmid‐based complementation assays. Crucially, reintroduction of the mutated *PIR2* allele into the Δ*pir2* strains restored the resistance phenotype in primarily mutated strains and even conveyed extra resistance to primarily wild‐type strains. In the highly responsive strains cgl19‐1 and cgl36‐1, MICs were restored to 1 and 0.25 µg/mL, respectively. This allele‐specific functional rescue demonstrates that the *G149_I167del* mutation itself is possibly one determinant conferring caspofungin resistance, rather than a simple loss‐of‐function of the *PIR2* gene.

**TABLE 3:** Caspofungin MICs of original, deleted, and complemented strains.

Drug	Strains	Original Strain	Δ*pir2* Strain	Δ*pir2* Strain with *PIR2_G149_I167del*
Caspofungin	CBS138	0.06	0.06	0.12
	cgl19‐1	1	0.5	1
	cgl36‐1	0.12	0.06	0.25
	cgl100‐1	0.5	0.25	0.5
	cgl121	0.12	0.12	0.5
Micafungin	CBS138	0.015	0.015	0.015
	cgl19‐1	0.015	0.015	0.015
	cgl36‐1	0.015	0.015	0.015
	cgl100‐1	<0.008	<0.008	<0.008
	cgl121	0.015	0.015	0.015
Anidulafungin	CBS138	0.03	0.03	0.03
	cgl19‐1	<0.015	<0.015	<0.015
	cgl36‐1	0.12	0.12	0.12
	cgl100‐1	0.03	0.03	0.03
	cgl121	0.12	0.12	0.12

## 3 Conclusions

This study, for the first time, explores the multifaceted mechanism for caspofungin heteroresistance in *C. glabrata*. Evaluating 156 clinical strains from CHIF‐NET (2014‐2019 program), we demonstrated that this phenotype is a surprisingly prevalent (25%) trait, exhibiting strict temperature dependence and drug specificity. We identify the calcineurin pathway as its master regulator, demonstrating that heteroresistance serves as a reservoir for stable resistance and uncover a novel candidate determinant (*PIR2_G149_I167del*) through machine learning‐guided genomic analysis.

Crucially, we identified the calcineurin pathway as its master regulator, whose activity is both necessary and sufficient for the phenotype. Mechanistically, the primary HR mechanism in *C. glabrata* distinguishes itself from models in *C. albicans* and *C. neoformans*, where aneuploidy is a common driver [27, 42, 43]. In our cohort, a recurrent aneuploidy pattern (e.g., ChrJ amplification) was observed in only 3 of 59 descendants, while other aneuploidies were strain‐specific. This contrasts with models in other fungal pathogens and underscores a greater reliance on point mutations and signaling pathways in *C. glabrata*. The observation that exogenous calcium partially rescues heteroresistance even under calcineurin inhibition adds a new layer of complexity to our understanding of cell wall stress survival. Whether this reflects a parallel calcium‐activated pathway or enhanced populational stress resilience independent of calcineurin signaling remains to be determined, but it underscores the central role of calcium homeostasis in governing caspofungin heteroresistance. This is highly concordant with the established model where increased intracellular calcium activates calmodulin and subsequently the calcineurin phosphatase, a pathway now implicated in regulating antifungal tolerance and persistence [20].

Clinically, echinocandin resistance predominantly emerges under specific drug selection pressure, as evidenced by their strong association with prior echinocandin exposure [44, 45, 46]. Another non‐stable resistance phenotype, persistence, has been demonstrated to serve as a reservoir for the evolution of echinocandin resistance [46]. In our study, we provided direct experimental evidence that this calcineurin‐mediated HR state is not a dead‐end but functions as a direct reservoir for the emergence of stably resistant descendants (24/59, 40.68%). Notably, among the 24 descendant strains with elevated caspofungin MICs (≥ 0.5 µg/mL), 16 lacked any mutations in the canonical *FKS2* hotspot regions. This coincides with the former demonstration that caspofungin held an inherit lower ability to identify *FKS* mutations compared to anidulafungin and micafungin, with a 90% sensitivity and 3% specialty [44, 45]. While this indicates that resistance in these isolates is not driven by the established *FKS2* mechanism, it is premature to conclude the operation of entirely novel resistance determinants. The elevated MICs could stem from other adaptive changes, including the heteroresistance phenotype itself, or from undetected genetic alterations outside the sequenced regions. Employing an integrated genomic and machine‐learning approach, we identified a novel mutation, an in‐frame deletion in the cell wall gene *PIR2* (*PIR2_G149_I167del*), as a high‐ranking candidate associated with resistance, which may operate alongside canonical *FKS2* mutations such as *FKS2_F659del*. Its modest effect is consistent with the emerging understanding of *PIR* family biology. *C. glabrata* harbors five *PIR* paralogs (*PIR1*–*PIR5*) [47], all annotated as orthologs of *Neurospora crassa* NCU04033—a protein shown to function as a cell wall stabilizer with significant functional redundancy among family members [48]. The presence of four additional *PIR* paralogs likely compensates for the loss of *PIR2*, explaining the mild phenotype observed. What is important is that its identification is a proof‐of‐concept that our data‐driven method can effectively surface new biological candidates from complexity for future functional investigation. This establishes a powerful paradigm for uncovering the genetic architecture of adaptive traits.

Our findings position caspofungin heteroresistance in *C. glabrata* as a phenotypically and mechanistically distinct entity. One of its defining features, strict temperature dependence (37°C), echoes one of the characteristics of tolerance, a non‐genetic, whole‐populational capacity to survive transient drug exposure, which has also been shown to be potentiated at host physiological temperature [33]. This phenotypic overlap suggests that heteroresistance and tolerance may share stress‐response signaling pathways, such as calcineurin, in response to cell wall stress. However, heteroresistance is distinguished by its subpopulation architecture and its role as a proven reservoir for stable genetic resistance, as demonstrated herein. Collectively, these features reveal a critical technical limitation: standard antifungal susceptibility testing, conducted at sub‐physiological temperatures and focused on population‐level MICs, may fail to detect this clinically relevant, drug‐specific adaptive reservoir, potentially leading to an underestimation of treatment failure risk.

Our study, specific in caspofungin, contrasts with the micafungin‐associated heteroresistance reported in *C. parapsilosis* [49], highlighting how the heteroresistance landscape is shaped by both the specific drug and the pathogen species. Consequently, the prevalent use of caspofungin in a given setting may select for caspofungin‐heteroresistant *C. glabrata*, whereas micafungin‐predominant pressure or intrinsic species factors can lead to micafungin heteroresistance [49]. The drug specificity further underscores its unique mechanism, which is partly rooted in their differential interaction with the drug target within the altered membrane environment resulting from regulated sphingolipid metabolism [50], thereby leading to the observed phenotype where our strains exhibited robust heteroresistance to caspofungin, they remained susceptible to micafungin and anidulafungin. Molecularly, although echinocandins share the Fks subunit target, subtle yet critical differences in their chemical structures, serum protein binding, and interaction with the enzyme's binding pocket exist. These differences are reflected in how specific *FKS* hotspot mutations disproportionately increase the IC_50_ for one drug over another [51]. Furthermore, intrinsic genetic, structural, and binding variations in Fks enzymes across *Candida* species create distinct baseline susceptibilities and predispose certain species to develop heteroresistance under selective pressure from specific echinocandins [51].

Beyond signaling pathways and genetic mutations, recent evidence suggests that membrane lipid composition, particularly sphingolipid biosynthesis, may contribute to drug‐specific susceptibility profiles [50, 52, 53]. Notably, caspofungin stress itself can induce reactive oxygen species (ROS) accumulation [54], which has been linked to decreased susceptibility and may act as a driver of lipid peroxidation, further inferring the possible role of lipid metabolism in caspofungin stress response [46]. In *C. glabrata*, studies have demonstrated that mutations or stress‐induced alterations in sphingolipid biosynthesis can lead to paradoxically caspofungin‐reduced‐susceptibility but micafungin‐increased‐susceptibility, a form of drug‐specific susceptibility phenotype [50]. This lipid remodeling likely alters the membrane microenvironment of Fks1/2, modulating drug‐target interaction and stress signaling cascades [53]. Notably, sphingolipid metabolism itself is temperature‐sensitive [55], which could provide a plausible link to the host temperature‐dependent heteroresistance we observed. It is thus reasonable to hypothesize that lipid adaptations may interact with or even be coordinated by the calcineurin signaling pathway to promote survival under caspofungin pressure. This potential intersection between lipid metabolism and stress signaling represents an intriguing avenue for future investigation.

While our phenotypic observations align with known biology, direct multi‐omics interrogation reveals a more sophisticated, coordinated survival strategy. We propose a “cell cycle‐coupled target expression” model: the HR subpopulation actively maintains a cell cycle state that guarantees high‐level production of the essential drug target Fks. This model, supported by our RNA‐seq data showing upregulation of cell cycle genes, is powerfully consistent with the established paradigm of cell cycle‐regulated *FKS* expression. Notably, our findings align with the work of Gonzalez‐Jimenez et al., who used a short‐lived transcriptional reporter to definitively show that *FKS1* and *FKS2* expression peaks in S‐phase [56]. Thus, we posit that this subpopulation couples its proliferative capacity with the means to withstand drug pressure, representing a core survival strategy.

Despite the comprehensive nature of our study, several limitations should be considered. First, the clinical cohort, while multicenter, was assembled retrospectively and may not fully represent the broader population at risk for invasive *C. glabrata* infections. The absence of identified epidemiological predictors for heteroresistance, while a notable finding, warrants validation in prospective studies with detailed longitudinal patient data. Second, our in vitro isolation of descendant strains, while instrumental in delineating the molecular mechanism, may not fully recapitulate the complex host microenvironment. Third, although our genetic and machine‐learning data implicate the *PIR2_G149_I167del* deletion in conferring resistance, the precise biochemical function of the mutant Pir2 protein in cell wall remodeling and whether it interacts with the calcineurin pathway remain to be fully elucidated. Moreover, while our transcriptomic data suggest that Crz1 likely contributes to the heteroresistance transcriptional program through regulation of broader cell wall integrity pathways rather than direct *FKS2* activation, direct mapping of Crz1 targets (e.g., by ChIP‐seq) under caspofungin pressure is needed to fully delineate its regulatory role. Furthermore, our analysis of genetic interactions between *PIR2* and *FKS2* mutations is based on computational predictions rather than experimental epistasis; definitive validation would require constructing double mutants in a common genetic background, which is beyond the scope of the current study. Finally, our understanding of the calcineurin‐independent, calcium‐activated survival pathway is still nascent, and the molecular sensors and effectors of this parallel route may constitute an important target for future investigation.

Looking forward, this work opens several critical avenues for research aimed at curbing the global threat of antimicrobial resistance. The molecular components of the calcium‐dependent backup pathway represent a high‐priority target for drug discovery. Crucially, our research revealed a profound therapeutic vulnerability: the calcineurin‐mediated HR phenotype itself. This suggests that adjunctive therapies targeting this pathway could be developed to extinguish the reservoir of resistant cells before they evolve into untreatable infections. By shifting the strategy from treating full‐blown resistance to preventing its emergence, our findings provide mechanistic support for the development of targeted antifungal stewardship strategies, which could improve outcomes for patients at risk of invasive *C. glabrata* infections.

## 4 Methods

### 4.1 Strains and Growth Conditions

We collected 156 *C. glabrata* strains from CHIF‐NET program, from 2014 to 2019. All strains were identified as *C. glabrata* at the species level by matrix‐assisted laser desorption ionization‐time of flight mass spectrometry (MALDI‐TOF MS, Vitek MS, bioMérieux, France). Including criteria consists of persistent *C. glabrata* infections or recurrent infections under clinical caspofungin administration with in vitro susceptibility tests suggesting caspofungin non‐resistant (MIC ≤ 0.25). All *C. glabrata* strains were cultured on YPD Broth (A507023‐0250, Sangon Biotech, China) or on YPD Agar (A507022‐0250, Sangon Biotech, China), 37°C or 30°C, if necessary.

### 4.2 Broth Microdilution Assay

MIC (Minimum Inhibition Concentration) determination for nine common antifungals in *C. glabrata* was carried out according to the broth microdilution method outlined by CLSI document M27 [57]. To obtain reproducible MIC values for comparative purposes across strains, we used Sensititre YeastOne Colorimetric Antifungal Panel (01. YO10, OXOID, UK), and at least three biological replicates for each strain were tested. *Candida parapsilosis* ATCC 22019 and *Candida krusei* ATCC 6258 were used as quality control.

To specifically evaluate the impact of physiological temperature on caspofungin susceptibility, we performed customized broth microdilution assays at three temperatures: 35°C (as per the manufacturer's standard for commercialized kits), alongside the experimental conditions of 30°C and 37°C. The MIC was determined visually after at least 24 h as the lowest drug concentration that resulted in a prominent reduction (≥ 90%) of growth compared to the positive/drug‐free control. For every strain under every test circumstance, at least three biological replicates for each strain tested were conducted.

### 4.3 Heteroresistance Screening and Confirming

We evaluated the caspofungin heteroresistance using PAP assays [49, 58], which was conducted on YPD agar. The rich nutrient composition of YPD agar supports the outgrowth of slow‐growing subpopulations under drug pressure and allows for clear visualization and quantification of individual colonies. Briefly, we prepared YPD agar plates with serial caspofungin (HY‐17006, MedChemExpress, USA) concentrations of 0, 0.06, 0.125, 0.25, 0.5, 1, (and 2 µg/mL, in initial screening), serial anidulafungin (HY‐13553, MedChemExpress, USA) concentrations of 0, 0.03, 0.06, 0.12, 0.25, 0.5 µg/mL, and serial micafungin (HY‐16321, MedChemExpress, USA) concentrations of 0, 0.015, 0.03, 0.06, 0.12, 0.25 µg/mL. Pick a single colony in 10 mL YPD broth and incubate the fungal suspension at 37°C, 200 rpm for 12–16 h. Add 5 uL of the 10^1^ to 10^5^ serial dilutions of the suspension to the gradient concentration of the YPD agar plate. Due to the attenuated growth of *C. glabrata* under antifungal pressure, colonies were counted after incubation at 30°C or 37°C for up to 144 h, which extends beyond standard incubation periods to ensure accurate enumeration of slow‐growing resistant subpopulation. Heteroresistance was defined as the ability of a strain to grow (≥ 2 colonies) on caspofungin at a concentration of either 0.5 µg/mL (for strains with MIC ≤ 0.06 µg/mL) or eight times of the MIC for the most sensitive cells (for strains with MIC ≥ 0.125 µg/mL). confirmed in three consecutive or four out of six biological replicates. For quantitative analysis, growth was measured as the log_10_(CFU/mL) derived from the original fungal suspension, which served as the y‐axis value for constructing the PAP curves.

### 4.4 Time‐Kill Assay

It is important to note that while PAP on YPD media can detect various drug‐surviving populations, the phenotype of heteroresistance is distinguished from general drug tolerance by its resistant subpopulation architecture and the capacity for proliferative rebound under continuous drug pressure [8]. This dynamically assesses the fungicidal activity of caspofungin against heteroresistant populations over time. Overnight cultures of test strains were diluted 1:100 in fresh YPD broth (10 mL), with or without drugs, calcium chloride, or calcineurin inhibitors, to initiate the assay. At designated time intervals (0, 8 if necessary, 24, 48, 72, 96, and 120 h), 0.5 mL aliquots were withdrawn from the cultures. The cells were pelleted by centrifugation (8000 rpm, 1 min), thoroughly washed, and resuspended in 0.5 mL of PBS buffer to eliminate carryover drug effects. The resulting suspensions were then subjected to serial ten‐fold dilution and spotted onto YPD agar for colony enumeration. The number of viable colonies was counted after 24 h of incubation at 37°C, with all assays performed in at least three biological duplicates to ensure reproducibility.

### 4.5 Transcriptomic Sequencing and Analysis

To delineate the transcriptional dynamics underpinning caspofungin stress response, we performed RNA sequencing on 5 heteroresistant strains under escalating caspofungin pressure (0, 0.125, and 1 µg/mL) for 144 h, and on three non‐heteroresistant strains (including CBS138) treated with caspofungin at 0 and 0.125 µg/mL for equally 144 h. Three biological replicates of each strain were subjected. Cell pellets were then snap‐frozen in liquid nitrogen and stored at −80°C before sequencing. We then employed Weighted Gene Co‐expression Network Analysis (WGCNA) on 5 heteroresistant strains to identify gene modules correlated with drug pressure. One key module, significantly associated with increasing caspofungin concentration, encompassed 248 genes. Genes that were upregulated in the three non‐heteroresistant (NonHR) strains under drug pressure were excluded from this module. Hub genes within the drug concentration‐correlated module were defined based on module membership (MM > 0.8 or < −0.8) and gene significance (GS > 0.6 or < −0.6). Functional enrichment analysis of these hub genes was performed using R (version 4.2.3) (https://cran.r‐project.org). Protein‐protein interaction (PPI) analysis was constructed to understand the functional relationships among these hub genes using STRING (https://cn.string‐db.org/). Using the Cytoscape (version 3.10.4) and it cytoHubba plugin [59, 60], we completed visualization and ranked genes by their network connectivity and identified the top hub genes within the PPI network.

### 4.6 Assessment of Stress‐Related Compounds

To systematically evaluate whether calcium signaling pathway contribute to caspofungin heteroresistance, we performed PAP assays and time‐kill assays in the presence of various compounds, including calcium chloride (CaCl_2_, 200 mm in agar plates and 100 mm in liquid medium; JF6365‐100 mL, JSENB, China) and calcineurin inhibitors FK506 (Tacrolimus, 10 µm in agar plates and 5 µm in liquid medium; 104987‐11‐3, Aladdin, China) and cyclosporin A (CsA, 20 and 10 µm in liquid medium; SC5120‐20 mg, Solarbio, China) [29, 61]. For PAP assays, these compounds were incorporated into YPD agar plates containing serial dilutions of caspofungin; parallel time‐kill assays were performed in liquid medium containing the same compounds. Considering the potential differences in drug activity between solid and liquid matrices, the concentrations used in liquid medium were halved relative to those in agar plates to ensure comparability. All assays were performed in at least three independent biological replicates.

### 4.7 Hereditary Stability Assay

The stability of the heteroresistant phenotype in three selected *C. glabrata* clinical isolates (cgl52, medium heteroresistant; cgl121, high heteroresistant; cgl138, mild heteroresistant) was evaluated based on Zhai et al. with a little modification [49]. Briefly, overnight cultures of each isolate, after adjusted primary OD_600_ to 2.0, were subcultured (1:100) into YPD broth containing 0.5 µg/ml caspofungin and incubated at 37°C, 200 rpm for 48 h. Subsequently, these cultures were passaged in the absence of drug pressure by a 1:1000 dilution into fresh YPD broth and grown for 72 h at 37°C, 200 rpm. Following each passage, cultures were serially diluted and plated onto both drug‐free YPD agar and YPD agar containing 0.5 µg/ml caspofungin. The colonies were counted after incubation at 37°C, 24 h, and the proportion of cells retaining resistance (CFUs from YPD agar plates with caspofungin/CFUs from drug‐free YPD agar plates) was calculated. This assay allows for the determination of whether the observed resistance is a stable trait or is lost in the absence of antifungal selection pressure.

### 4.8 Gene Knock‐Out and Complementation

Gene knock‐out was carried out by homologous recombination mediated transformation, and targeted genes were replaces by nourseothricin (NAT) resistance cassette [62], which was amplified from plasmid *pCN‐Lys21* [29]. The *C. glabrata* orthologue of a *S. cerevisiae* gene is identified by blast search (http://candidagenome.org/) and checking its chromosomal context (http://www.candidagenome.org/download/sequence/C_glabrata_CBS138). Design four pairs of primers for amplification of upstream amplification, NAT resistance cassette, downstream amplification, and verification, respectively. Specific sequences of primers used can be seen in Table S10. After amplified, fused together, confirmed and purified, about 1 µg of construct sequence was then transformed into 50–55 µL of lithium‐acetate‐method‐based competent *C. glabrata* cells using electroporation (1500 V, 25 µF, 200Ω). Pick single colonies from 400 µg/mL nourseothricin sulfate (96736‐11‐7, ACMEC Biochemical, China)‐containing plates and confirm with *checkF* and *checkR* primers. When the electrophoresis banding was of the correct length and confirmed by Sanger sequencing, the knockout construct was successfully constructed.

Plasmid complementation was used for knock‐out‐gene reintroducing, of which we chose shuttle plasmid *pCN‐Lys21* and replace the NAT resistance cassette to hygromycin B resistance cassette [63]. The electroporation protocol is the same as described above. The success of the introduction was judged by visible colony growth on 800 mg/L hygromycin B (31282‐04‐9, ACMEC Biochemical, China) ‐containing YPD agar plate, correct AGE electrophoretic banding, and Sanger sequencing of the PCR product using *plasmid‐checkF* and *plasmid‐checkR* primers. The primers used are also listed in Table S10.

### 4.9 In Vitro Challenge and Selection of Descendant Strains

To determine the fate of heteroresistant populations under high‐dose caspofungin pressure, we subjected all 39 HR isolates to YPD agar plates containing 1 µg/mL caspofungin. However, due to varying degrees of heteroresistance, four strains (cgl7, cgl103, cgl108, cgl132) showed no progeny colonies on the plates. After 144 h at 37°C, 59 descendant strains were randomly selected and harvested. They were subsequently cultured in caspofungin‐containing YPD broth (0.5 µg/mL) for propagation and then subjected to antifungal susceptibility testing and whole‐genome sequencing.

### 4.10 Susceptibility Stability Assay

To assess the genetic stability of caspofungin resistance in clones directly isolated from heteroresistant populations, we performed serial passaging in drug‐free medium. Two types of progeny were evaluated: primary clones picked directly from YPD agar plates containing 1 µg/mL caspofungin during the initial PAP screening (designated PAP‐derived clones), and secondary clones obtained after subsequent liquid culture of PAP‐derived clones in YPD broth containing 0.5 µg/mL caspofungin for at least 96 h (designated descendant clones), which corresponded to the strains subjected to whole‐genome sequencing. For each clone, a single colony was streaked onto drug‐free YPD agar and incubated at 37°C for 24 h. This process was repeated for five consecutive passages. After all the passages, the caspofungin MIC of the passaged population was determined using the broth microdilution method as described above. Stability was defined as the absence of a ≥ 2‐fold change in MIC compared to the original clone prior to passaging.

### 4.11 Genomic Characterization and Mutation Profiling

To identify genetic alterations associated with drug resistance, we performed whole‐genome sequencing analysis on the paired parent‐descendant isolates. Sequencing reads were aligned to the *C. glabrata* CBS138 reference genome (http://www.candidagenome.org/download/sequence/C_glabrata_CBS138) using BWA‐MEM and processed following standard practices, including duplicate removal with Picard. Variant calling for SNPs and indels was conducted using the GATK HaplotypeCaller pipeline, and the resulting variants were functionally annotated with SnpEff.

### 4.12 Multilocus Sequence Typing and Phylogenetic Analysis

Multilocus sequence types (STs) were determined in silico from the whole‐genome sequencing data. The assembled contigs, generated by SPAdes, were analyzed using the MLST software (https://github.com/tseemann/mlst) to assign genome‐based types [64]. To elucidate the phylogenetic relationships among the isolates, a maximum‐likelihood tree was reconstructed with IQ‐TREE [65]. The best‐fit nucleotide substitution model (TVM + F + R2) was first selected using ModelFinder based on the Bayesian Information Criterion. The tree was then inferred with high statistical support, employing 1000 ultrafast bootstrap replicates. The final phylogenetic tree was visualized and annotated using the Interactive Tree of Life (iTOL) platform [66].

### 4.13 Machine Learning‐Based Gene Selection

To systematically identify genetic determinants contributing to caspofungin resistance, we employed a supervised machine learning framework [67]. The magnitude of resistance was modeled as a continuous phenotype, defined as log_2_[MIC_descendant/MIC_parent] (STATUS). A binary feature matrix was constructed from whole‐genome sequencing data, wherein each mutation was encoded for its presence or absence in each isolate.

We first addressed the high dimensionality of the genomic data through a multi‐stage feature selection process. Features exhibiting minimal correlation with the resistance phenotype (absolute Pearson correlation < 0.05) were initially filtered. Subsequently, the Boruta algorithm, a robust wrapper method based on Random Forest, was applied to identify a set of statistically significant mutations by comparing their importance against randomized shadow features over multiple iterations [68, 69]. To further ensure the stability of the selected features, we performed stability selection using Lasso regression across 20 bootstrap samples, retaining only those mutations consistently selected in most iterations [70, 71]. The convergence of these complementary filtering strategies yielded a refined and robust set of genetic variants for predictive modeling.

We then trained three distinct regression models to capture the complex relationships between the selected mutations and the resistance phenotype. A Random Forest model was optimized to capture non‐linear interactions and complex dependencies [72]. An Extreme Gradient Boosting (XGBoost, tuned for tree depth (3–4), learning rate (0.01–0.1), number of rounds (100–150), and subsampling/column‐sampling rates (0.8)) model was tuned to sequentially correct prediction errors, providing high predictive performance with careful regularization to prevent overfitting [73, 74]. Finally, an Elastic Net regression model was employed to handle potential multicollinearity among features while encouraging a sparse solution [75]. All models were rigorously tuned via 5‐fold cross‐validation on the training set, with the root‐mean‐square error (RMSE) serving as the primary optimization metric.

To leverage the complementary strengths of the individual models and enhance predictive robustness, we implemented a dynamic weighted ensemble strategy. The final prediction was generated as a weighted average of the predictions from the three base models, where each model's weight was dynamically assigned based on its inverse RMSE performance on the independent test set. Feature importance was extracted from each model using model‐specific metrics: permutation importance for Random Forest, gain‐based importance for XGBoost, and absolute coefficient values for Elastic Net. These importance scores were standardized and aggregated into a composite importance score, weighted by the corresponding model's performance weight in the ensemble. This composite ranking prioritizes mutations that are consistently deemed important by multiple, well‐performing algorithms, thereby providing a more reliable and biologically interpretable list of candidate resistance determinants. The final ensemble model was evaluated on a held‐out test set using standard regression metrics, including the coefficient of determination (R^2^) and RMSE.

### 4.14 Data Analysis

All statistical analyses were performed using R (version 4.2.3; https://cran.r‐project.org) and GraphPad Prism (version 10.0, GraphPad Software, San Diego, CA, USA). Continuous variables (e.g., patient age) were assessed for normality using Shapiro‐Wilk tests and visual inspection of Q‐Q plots. Normally distributed data were expressed as mean ± standard deviation and compared using Student's t‐tests, while non‐normally distributed data were presented as median with interquartile range and analyzed using Mann‐Whitney U tests. Categorical variables (including gender, department classification, specimen type, and sequence type) were summarized as frequencies and percentages, with associations evaluated using Pearson's chi‐square tests or Fisher's exact tests when expected variable frequencies were below 5. For sequence type analysis, we performed both overall chi‐squares testing of distribution and supplementary binomial tests for individual sequence types with adequate sample size (n ≥ 5), comparing their observed heteroresistance rates against the overall population prevalence of 25.0%. All statistical tests were two‐tailed, and a *p*‐value < 0.05 was considered statistically significant.

## Author Contributions

Y.S. and Y.L. (Yingxing Li) conceived and designed the study. Y.S. performed the experimental work and wrote the original manuscript. Y.L. (Yi Li) collected clinical samples, conducted the bioinformatic analysis, and contributed to data interpretation. M.X., D.G., D.S.P., E.S., and Y.X. provided intellectual advice, reviewed, and edited the manuscript. Y.X., Y.L. (Yingxing Li), M.X., and Y.L. (Yi Li) acquired funding and supervised the project. All authors have read and agreed to the published version of the manuscript.

## Funding

This work was supported by the National Science and Technology Major Project [No. 2024ZD0532800]; the Fundamental Research Funds for the Central Universities, Peking Union Medical College [3332025024]; National High Level Hospital Clinical Research Funding [2025‐PUMCH‐C‐044]; Peking Union Medical College Hospital Talent Cultivation Program (Category C) (UBJ11583).

## Ethics Statement

The study was conducted in accordance with the Declaration of Helsinki and approved by the Human Research Ethics Committee of Peking Union Medical College Hospital (No. S‐263). Informed consent was obtained from all subjects involved in the study.

## Conflicts of Interest

The authors declare no conflicts of interest.

## Supporting information




**Supporting File**: advs76369‐sup‐0001‐SuppMat.docx.

## Data Availability

All short‐read data were uploaded to the NCBI under the project number PRJNA1010673 (part of previously published clinical isolates) and PRJNA1371698 (newly added data).
