# Supplementary material for: Calcineurin‐Dependent Stress Adaptation Enables Caspofungin Heteroresistance Leading to Stable Resistance in Candida Glabrata
Source: Adv Sci (Weinh). 2026 Jun 28:e76369. Online ahead of print. doi: 10.1002/advs.76369 (PMC13336451; doi:10.1002/advs.76369)
Supplement: Supplementary file 1 — Supporting File: advs76369‐sup‐0001‐SuppMat.docx. [file ADVS-9999-e76369-s001.docx]

Supporting Information

**Fig. S1.**


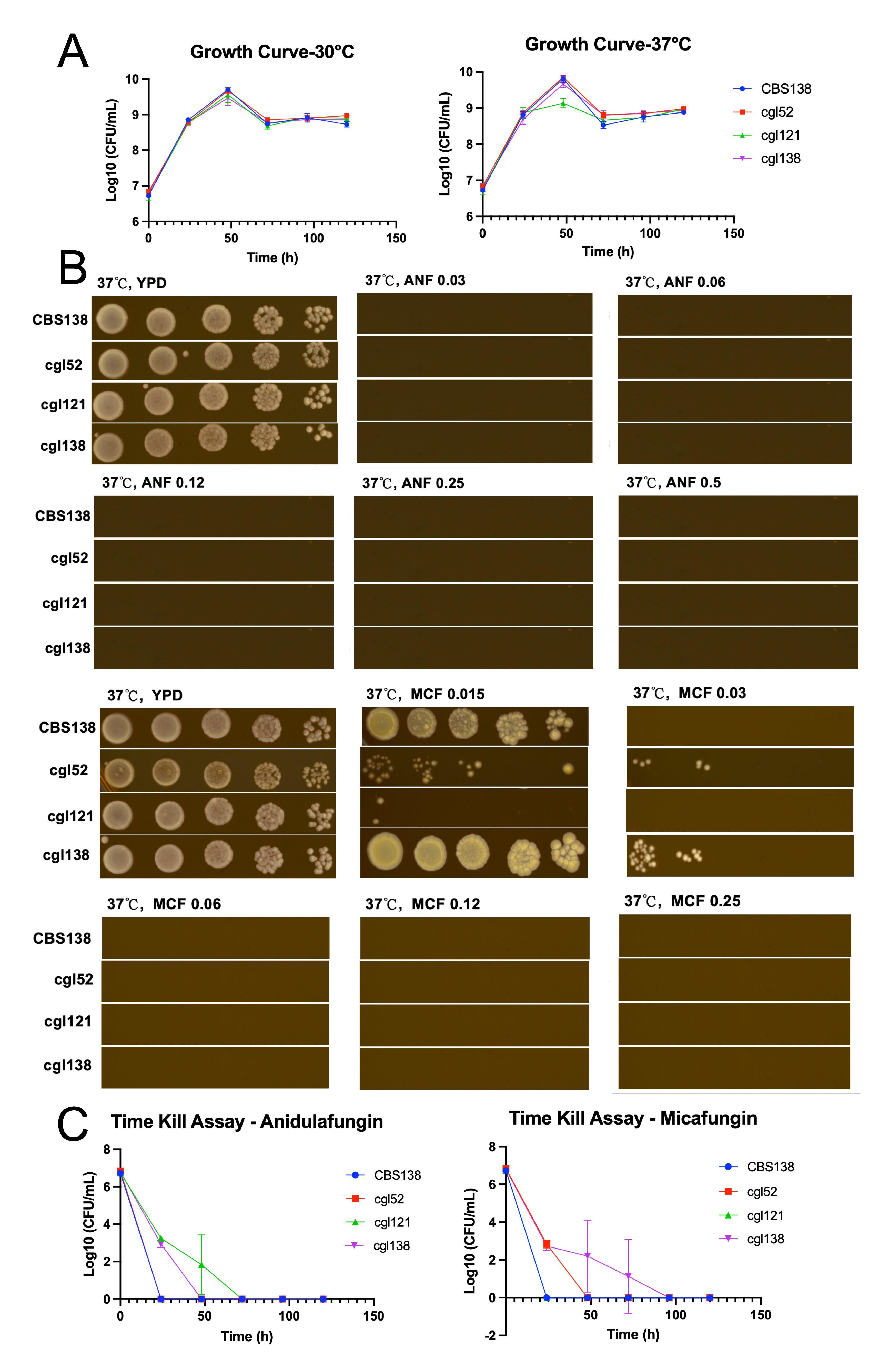


**Figure S1. Heteroresistance is a Drug-Specific Feature of Caspofungin.** (A) Drug-free growth curve of HR strains and CBS138 under 30°C and 37°C. (B) Spot assays of caspofungin HR strains under serial concentrations of micafungin and anidulafungin. (C) Time-kill assays of HR strains under 0.5 µg/mL micafungin and 0.25 µg/mL anidulafungin.

Fig. S2.


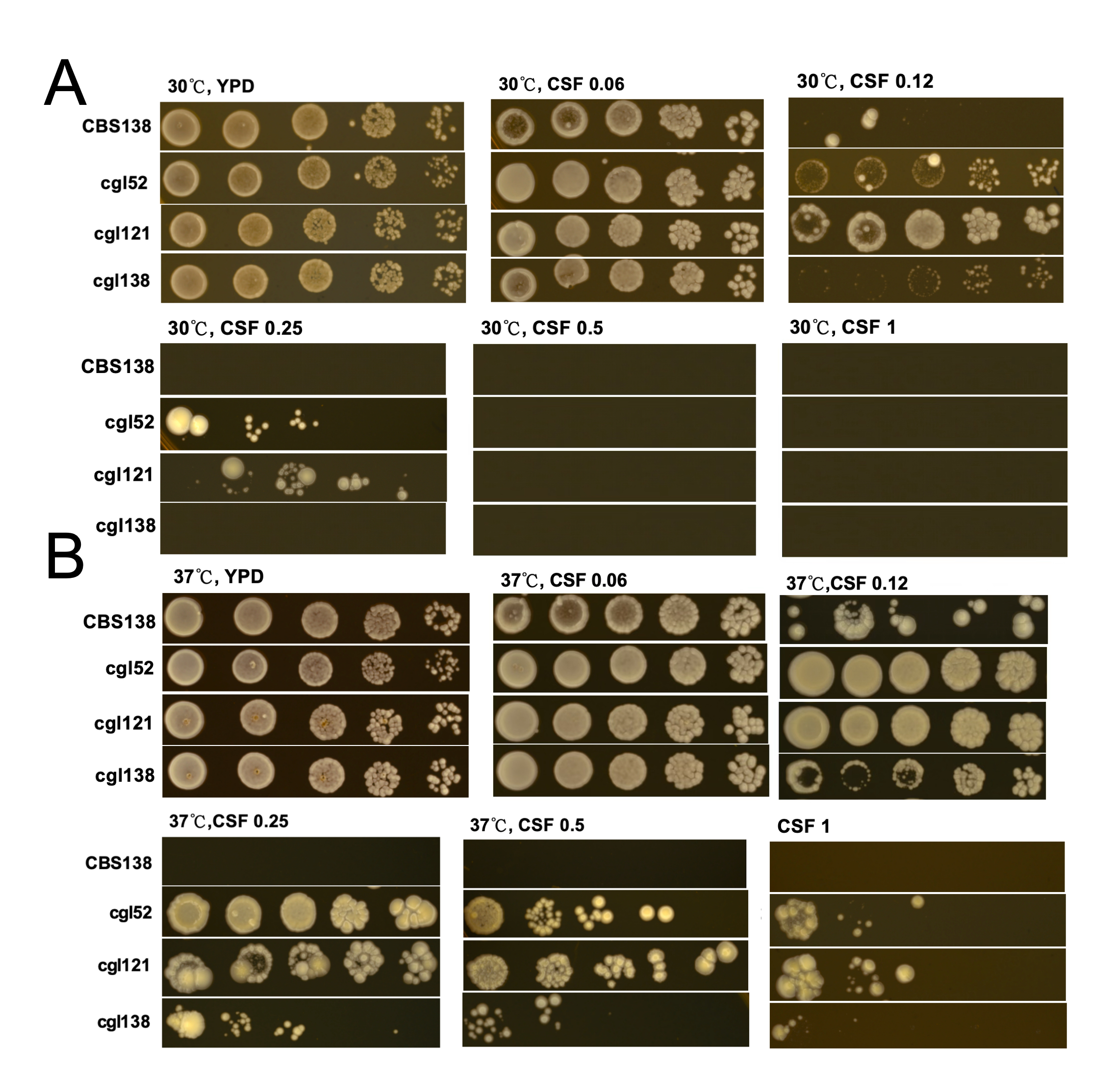


**Figure S2. Heteroresistance is a Temperature-Dependent Feature.** (A) Population analysis profiling of HR strains under serial concentrations of caspofungin, 37°C. (B) Population analysis profiling of HR strains under serial concentrations of caspofungin, 30°C.

Fig. S3.


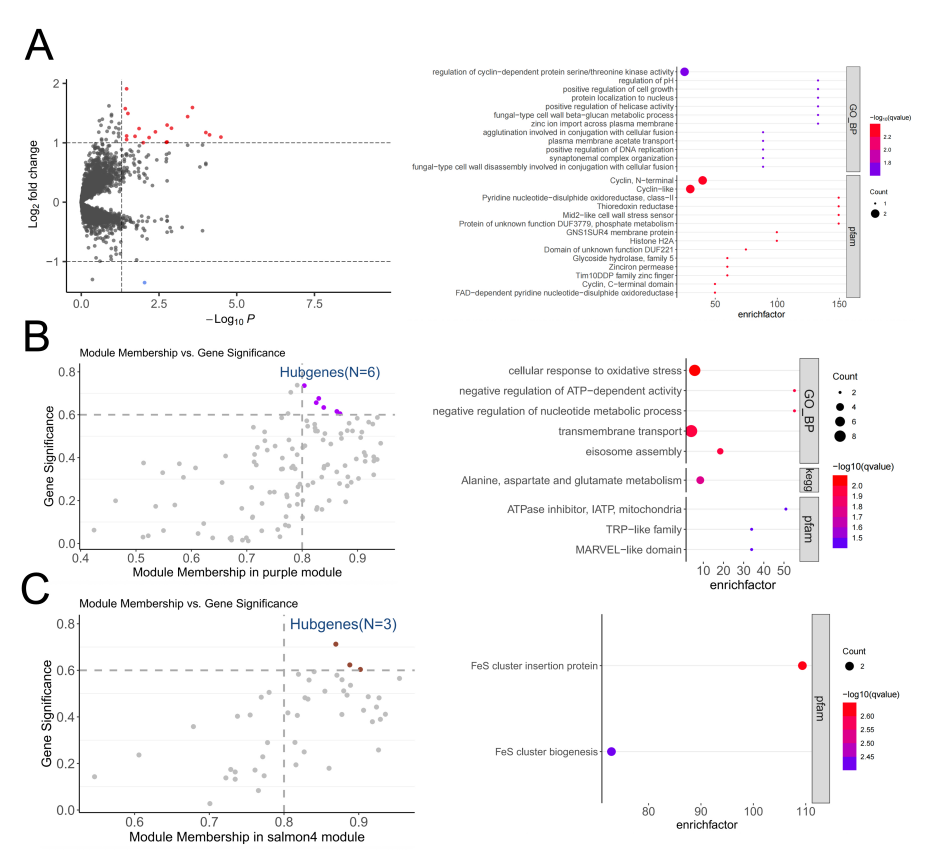


**Figure S3. Transcriptional features reveal core functional modules in heteroresistance.** (A) Volcano plot and functional analysis between low-caspofungin-concentration and caspofungin-free group in nonHR strains. (B) Illustration of the PURPLE module and functional enrichment analysis of the hub genes. The top significantly enriched terms are shown, highlighting cellular oxidative stress and transmembrane transport as two of the core adaptive processes. (C) Illustration of the SALMON module and functional enrichment analysis of the hub genes. The top significantly enriched terms are shown, highlighting FeS cluster insertion protein as the core adaptive processes.

Fig. S4.


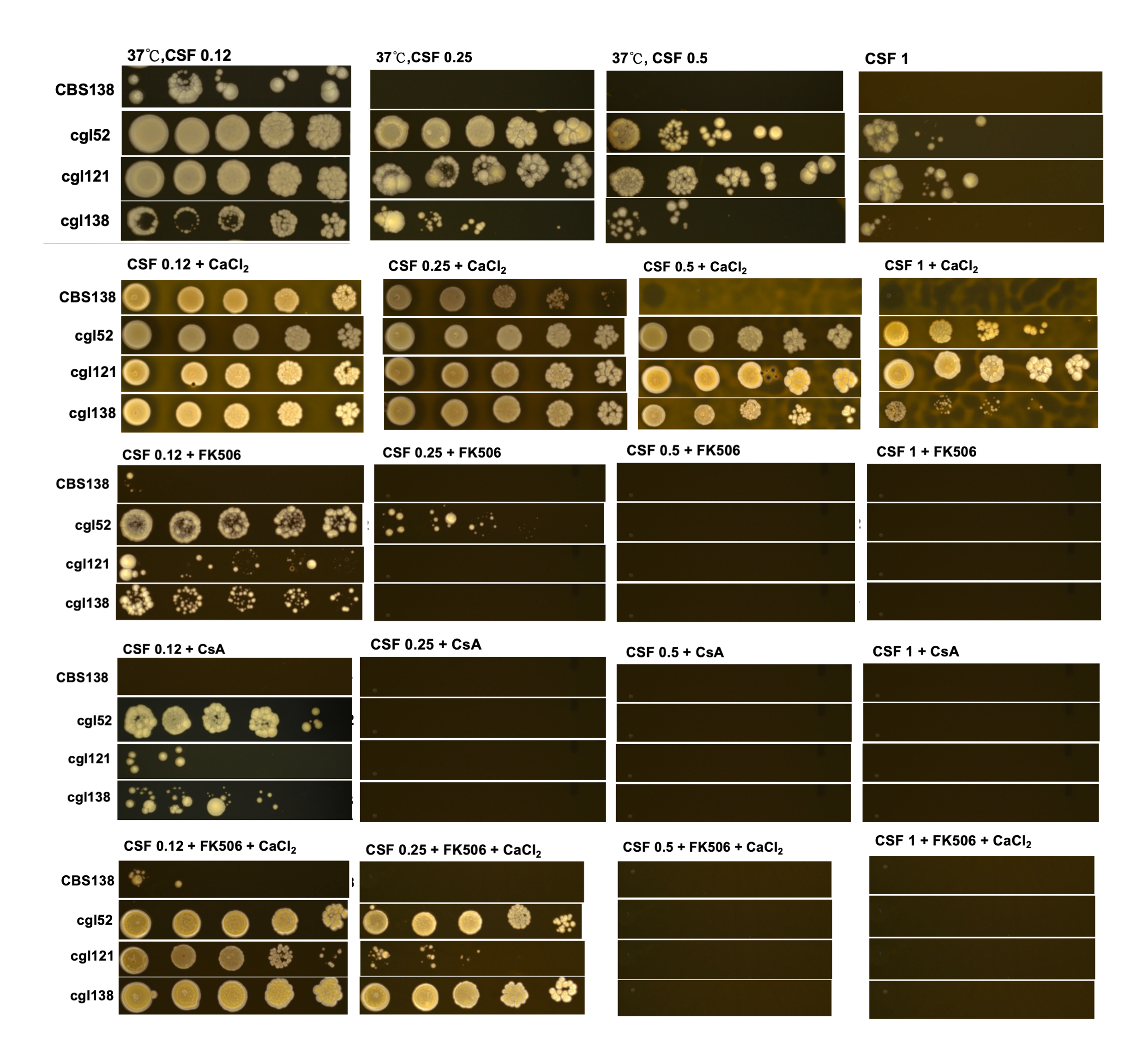


**Figure S4. HR Phenotype is Rescued by CaCl_2_ and Abolished by FK506.**Population analysis profiling of HR strains under CaCl_2_, FK506, CsA and combination of CaCl_2_ + FK506, all under caspofungin of concentrations from 0.125 µg/mL to 1 µg/mL.

Table S1. Epidemiology Information of 156 *N. glabratus* Strains Included

| Phenotype | Strains | STs | geo | Age | Gender | Department | Type | Caspofungin MICs |
| --- | --- | --- | --- | --- | --- | --- | --- | --- |
| HR | cgl7 | ST7 | Henan | 50 | female | ICU | Blood | 0.03 |
|  | cgl9 | ST7 | Shandong | 33 | female | Obstetrics | Blood | 0.12 |
|  | cgl10 | ST7 | Shandong | 25 | female | Hepatobiliary (and Pancreatic) Surgery | Blood | 0.06 |
|  | cgl12 | ST7 | Shandong | 60 | female | Metabolic Diseases Department | Blood | 0.03 |
|  | cgl18 | ST26 | Guangdong | 70 | male | ICU | Blood | 0.06 |
|  | cgl19 | ST7 | Guangdong | 43 | female | Nephrology | Blood | 0.06 |
|  | cgl26 | ST7 | Yunnan | 64 | male | ICU | Blood | 0.06 |
|  | cgl27 | ST7 | Yunnan | 63 | male | ICU | Drainage Fluid | 0.12 |
|  | cgl28 | ST55 | Hebei | 88 | male | ICU | Blood | 0.12 |
|  | cgl34 | ST7 | Fujian | 74 | female | Hepatobiliary (and Pancreatic) Surgery | Blood | 0.03 |
|  | cgl36 | ST7 | Zhejiang | 31 | female | Hepatobiliary (and Pancreatic) Surgery | Ascite | 0.06 |
|  | cgl41 | ST22 | Heilongjiang | 47 | female | Gynecology | Secretion | 0.03 |
|  | cgl43 | ST10 | Heilongjiang | 44 | female | Gynecology | Secretion | 0.12 |
|  | cgl44 | ST7 | Heilongjiang | 90 | male | vip2 | Blood | 0.06 |
|  | cgl45 | ST55 | Hunan | 46 | male | Neurology Department | Blood | 0.06 |
|  | cgl52 | ST7 | Hebei | 87 | male | ICU | Ascite | 0.06 |
|  | cgl55 | ST208 | Chongqing | 47 | female | Dermatology | Secretion | 0.06 |
|  | cgl59 | ST7 | Henan | 51 | female | Infectious Diseases Department | Blood | 0.06 |
|  | cgl61 | ST10 | Liaoning | 60 | male | ICU | Blood | 0.12 |
|  | cgl67 | ST7 | Beijing | 87 | male | ICU | Blood | 0.03 |
|  | cgl69 | ST3 | Beijing | 90 | male | ICU | Drainage Fluid | 0.06 |
|  | cgl74 | ST3 | Liaoning | 64 | female | Gynecology | Blood | 0.06 |
|  | cgl84 | ST195 | Fujian | 60 | female | ICU | Blood | 0.03 |
|  | cgl94 | ST7 | Jiangsu | 47 | male | ICU | Lavage Fluid | 0.12 |
|  | cgl99 | ST7 | Jiangsu | 60 | female | ICU | Pus | 0.03 |
|  | cgl100 | ST7 | Jiangsu | 40 | female | ICU | Lavage Fluid | 0.03 |
|  | cgl102 | ST7 | Jiangsu | 27 | male | Pulmonology | Blood | 0.06 |
|  | cgl103 | ST10 | Jiangsu | 67 | male | Hepatobiliary (and Pancreatic) Surgery | Blood | 0.03 |
|  | cgl106 | ST7 | Jiangsu | 66 | male | Emergency | Blood | 0.12 |
|  | cgl108 | ST10 | Jiangsu | 38 | male | General Surgery | Blood | 0.03 |
|  | cgl113 | ST7 | Zhejiang | 71 | female | General Surgery | Blood | 0.03 |
|  | cgl121 | ST7 | Beijing | 91 | male | Pulmonology | Lavage Fluid | 0.12 |
|  | cgl132 | ST7 | Chongqing | 92 | male | ICU | Blood | 0.12 |
|  | cgl138 | ST55 | Sichuan | 65 | male | ICU | Catheter | 0.12 |
|  | cgl152 | ST3 | Gansu | 55 | male | General Surgery | Drainage Fluid | 0.06 |
|  | cgl160 | ST26 | Guangdong | 67 | male | ICU | Tissue | 0.12 |
|  | cgl164 | ST10 | Shanghai | 60 | male | Gastroenterology | Drainage Fluid | 0.12 |
|  | cgl176 | ST7 | Liaoning | 88 | male | ICU | Ascite | 0.12 |
|  | cgl180 | ST7 | Xinjiang | 80 | female | Pulmonology | Blood | 0.12 |
| NonHR | cgl1 | ST7 | Hebei | 63 | female | Infectious Diseases Department | Blood | 0.06 |
|  | cgl2 | ST43 | Hebei | 50 | male | Orthopedics Department | Blood | 0.06 |
|  | cgl3 | ST7 | Hubei | 39 | male | Urology Department | Blood | 0.06 |
|  | cgl4 | ST7 | Henan | 78 | female | Orthopedics Department | Puncture Fluid | 0.12 |
|  | cgl5 | ST7 | Henan | 49 | female | Oncology Department | Blood | 0.06 |
|  | cgl8 | ST7 | Shandong | 73 | male | ICU | Blood | 0.06 |
|  | cgl15 | ST203 | Guangdong | 10 | male | General Surgery | Drainage Fluid | 0.03 |
|  | cgl17 | ST19 | Guangdong | 68 | male | Gastrointestinal Surgery | Drainage Fluid | 0.06 |
|  | cgl20 | ST8 | Guangdong | 5 | male | Pediatric Surgery | Blood | 0.015 |
|  | cgl21 | ST19 | Guangdong | 75 | male | ICU | Drainage Fluid | 0.06 |
|  | cgl23 | ST19 | Guangdong | 45 | male | Organ Transplantation Department | Drainage Fluid | 0.12 |
|  | cgl24 | ST10 | Yunnan | 39 | female | Gynecology | Blood | 0.03 |
|  | cgl25 | ST7 | Yunnan | 41 | male | Gastrointestinal Surgery | Blood | 0.06 |
|  | cgl29 | ST7 | Hebei | 56 | male | ICU | Ascite | 0.12 |
|  | cgl30 | ST7 | Hebei | 74 | female | ICU | Blood | 0.015 |
|  | cgl31 | ST7 | Fujian | 54 | male | Urology Department | Blood | 0.06 |
|  | cgl32 | ST3 | Fujian | 56 | female | General Surgery | Blood | 0.03 |
|  | cgl33 | ST3 | Fujian | 56 | male | Gastroenterology | Blood | 0.12 |
|  | cgl35 | ST7 | Zhejiang | 50 | male | Hepatobiliary (and Pancreatic) Surgery | Blood | 0.06 |
|  | cgl38 | ST3 | Beijing | 28 | female | Pediatric Surgery | Blood | 0.06 |
|  | cgl39 | ST7 | Beijing | 53 | female | Emergency | Blood | 0.03 |
|  | cgl46 | ST7 | Hunan | 63 | male | Geriatrics Department | Blood | 0.03 |
|  | cgl47 | ST22 | Hunan | 55 | male | Pulmonology | Blood | 0.03 |
|  | cgl48 | ST7 | Tianjin | 88 | male | Infectious Diseases Department | Blood | 0.06 |
|  | cgl49 | ST3 | Tianjin | 84 | male | ICU | Blood | 0.03 |
|  | cgl50 | ST7 | Tianjin | 94 | male | General Surgery | Blood | 0.06 |
|  | cgl51 | ST7 | Hebei | 76 | female | ICU | Blood | 0.06 |
|  | cgl53 | ST7 | Hebei | 62 | male | ICU | Ascite | 0.06 |
|  | cgl54 | ST10 | Chongqing | 72 | female | Cardiovascular Surgery | Catheter | 0.06 |
|  | cgl58 | ST22 | Henan | 50 | female | ICU | Blood | 0.06 |
|  | cgl60 | ST7 | Henan | 68 | female | ICU | Catheter | 0.12 |
|  | cgl62 | ST7 | Liaoning | 58 | male | ICU | Blood | 0.06 |
|  | cgl64 | ST7 | Liaoning | 52 | female | Pulmonology | Catheter | 0.06 |
|  | cgl65 | ST7 | Liaoning | 58 | male | Emergency | Bile | 0.12 |
|  | cgl66 | ST7 | Liaoning | 65 | male | Hepatobiliary (and Pancreatic) Surgery | Catheter | 0.06 |
|  | cgl68 | ST7 | Beijing | 71 | male | Oncology Department | Blood | 0.06 |
|  | cgl70 | ST7 | Beijing | 47 | male | Hepatobiliary (and Pancreatic) Surgery | Drainage Fluid | 0.06 |
|  | cgl71 | ST22 | Liaoning | 76 | male | General Surgery | Blood | 0.03 |
|  | cgl72 | ST7 | Liaoning | 77 | female | ICU | Blood | 0.06 |
|  | cgl73 | ST3 | Liaoning | 67 | male | Nephrology | Blood | 0.12 |
|  | cgl75 | ST7 | Henan | 67 | male | Supportive Care | Secretion | 0.06 |
|  | cgl76 | ST7 | Henan | 66 | female | General Surgery | Ascite | 0.06 |
|  | cgl77 | ST7 | Henan | 38 | male | Nephrology | Blood | 0.03 |
|  | cgl79 | ST7 | Sichuan | 63 | male | Supportive Care | Blood | 0.06 |
|  | cgl80 | ST3 | Sichuan | 45 | female | ICU | Drainage Fluid | 0.06 |
|  | cgl81 | ST10 | Sichuan | 78 | female | ICU | Blood | 0.03 |
|  | cgl82 | ST7 | Fujian | 88 | male | ICU | Ascite | 0.03 |
|  | cgl83 | ST7 | Fujian | 74 | female | ICU | Blood | 0.03 |
|  | cgl85 | ST7 | Fujian | 76 | male | ICU | Blood | 0.06 |
|  | cgl86 | ST7 | Zhejiang | 77 | male | Supportive Care | Blood | 0.03 |
|  | cgl87 | ST7 | Zhejiang | 90 | male | ICU | Blood | 0.25 |
|  | cgl89 | ST10 | Zhejiang | 91 | male | ICU | Blood | 0.03 |
|  | cgl90 | ST7 | Xinjiang | 39 | female | ICU | Blood | 0.06 |
|  | cgl91 | ST7 | Gansu | 86 | male | Urology Department | Blood | 0.03 |
|  | cgl92 | ST45 | Gansu | 75 | male | ICU | Blood | 0.03 |
|  | cgl93 | ST7 | Gansu | 60 | male | Urology Department | Blood | 0.06 |
|  | cgl95 | ST7 | Jiangsu | 80 | female | General Internal Medicine | Lavage Fluid | 0.06 |
|  | cgl96 | ST7 | Jiangsu | 47 | female | Urology Department | Blood | 0.06 |
|  | cgl97 | ST10 | Jiangsu | 71 | male | Hepatobiliary (and Pancreatic) Surgery | Blood | 0.03 |
|  | cgl98 | ST7 | Jiangsu | 52 | male | Gastroenterology | Pus | 0.06 |
|  | cgl101 | ST7 | Jiangsu | 23 | female | Gastroenterology | Ascite | 0.03 |
|  | cgl104 | ST203 | Jiangsu | 15 | female | Emergency | Blood | 0.03 |
|  | cgl105 | ST195 | Jiangsu | 47 | male | ICU | Blood | 0.03 |
|  | cgl110 | ST7 | Jiangsu | 28 | male | General Surgery | Blood | 0.12 |
|  | cgl111 | ST45 | Sichuan | 47 | female | General Surgery | Ascite | 0.03 |
|  | cgl112 | ST19 | Sichuan | 43 | male | Tuberculosis Ward | Blood | 0.03 |
|  | cgl114 | ST3 | Zhejiang | 64 | male | ICU | Pleural Effusion | 0.03 |
|  | cgl117 | ST7 | Beijing | 65 | male | Oncology Department | Catheter | 0.06 |
|  | cgl120 | ST7 | Beijing | 68 | male | Emergency | Blood | 0.06 |
|  | cgl122 | ST45 | Beijing | 47 | female | Immunology Department | Blood | 0.015 |
|  | cgl124 | ST19 | Tianjin | 69 | male | Cardiovascular Surgery | Blood | 0.03 |
|  | cgl125 | ST10 | Tianjin | 89 | male | Geriatrics Department | Blood | 0.06 |
|  | cgl126 | ST7 | Ningxia | 54 | male | ICU | Blood | 0.06 |
|  | cgl127 | ST7 | Ningxia | 41 | male | Oncology Department | Blood | 0.06 |
|  | cgl128 | ST7 | Ningxia | 43 | male | Emergency | Catheter | 0.06 |
|  | cgl129 | ST7 | Ningxia | 71 | male | Hepatobiliary (and Pancreatic) Surgery | Bile | 0.12 |
|  | cgl130 | ST7 | Chongqing | 87 | male | ICU | Blood | 0.06 |
|  | cgl131 | ST7 | Chongqing | 49 | female | Urology Department | Blood | 0.06 |
|  | cgl133 | ST7 | Sichuan | 55 | female | Department of Integrated Traditional Chinese and Western Medicine | Blood | 0.06 |
|  | cgl134 | ST7 | Sichuan | 64 | female | ICU | Ascite | 0.12 |
|  | cgl135 | ST7 | Sichuan | 61 | female | ICU | Blood | 0.06 |
|  | cgl136 | ST19 | Sichuan | 42 | male | ICU | Blood | 0.12 |
|  | cgl137 | ST7 | Sichuan | 59 | female | Emergency | Blood | 0.06 |
|  | cgl139 | ST7 | Hubei | 53 | female | Emergency | Blood | 0.12 |
|  | cgl140 | ST19 | Hubei | 67 | female | ICU | Ascite | 0.12 |
|  | cgl141 | ST7 | Hebei | 48 | female | Infectious Diseases Department | Ascite | 0.12 |
|  | cgl142 | ST7 | Fujian | 43 | female | ICU | Blood | 0.12 |
|  | cgl143 | ST7 | Fujian | 72 | male | ICU | Blood | 0.12 |
|  | cgl144 | ST7 | Fujian | 74 | male | Oncology Department | Blood | 0.06 |
|  | cgl146 | ST45 | Gansu | 60 | female | General Surgery | Blood | 0.06 |
|  | cgl147 | ST7 | Gansu | 39 | male | General Surgery | Blood | 0.06 |
|  | cgl148 | ST15 | Gansu | 72 | female | General Surgery | Drainage Fluid | 0.12 |
|  | cgl149 | ST7 | Gansu | 78 | male | Interventional Pain Management Department | Blood | 0.12 |
|  | cgl150 | ST3 | Gansu | 49 | female | Gastroenterology | Ascite | 0.06 |
|  | cgl151 | ST7 | Gansu | 78 | female | General Surgery | Pus | 0.12 |
|  | cgl153 | ST7 | Shanghai | 40 | female | null | Blood | 0.06 |
|  | cgl154 | ST43 | Shanghai | 95 | male | Infectious Diseases Department | Blood | 0.12 |
|  | cgl155 | ST3 | Shanghai | 63 | female | General Surgery | Replacement Fluid | 0.06 |
|  | cgl156 | ST7 | Shanghai | 57 | male | General Surgery | Drainage Fluid | 0.06 |
|  | cgl157 | ST7 | Shanghai | 60 | female | Infectious Diseases Department | Lavage Fluid | 0.12 |
|  | cgl158 | ST10 | Shanghai | 56 | female | Gastroenterology | Ascite | 0.03 |
|  | cgl159 | ST7 | Guangdong | 65 | female | General Internal Medicine | Blood | 0.06 |
|  | cgl161 | ST304 | Guangdong | 62 | male | ICU | Blood | 0.12 |
|  | cgl162 | ST22 | Jilin | 82 | female | ICU | Drainage Fluid | 0.06 |
|  | cgl163 | ST55 | Jilin | 50 | female | Hepatobiliary (and Pancreatic) Surgery | Blood | 0.06 |
|  | cgl165 | ST7 | Shanghai | 50 | male | General Surgery | Blood | 0.06 |
|  | cgl166 | ST7 | Shanghai | 47 | female | Urology Department | Puncture Fluid | 0.06 |
|  | cgl167 | ST3 | Shanghai | 65 | male | General Surgery | Blood | 0.06 |
|  | cgl168 | ST10 | Shanghai | 56 | male | General Surgery | Blood | 0.06 |
|  | cgl169 | ST15 | Shanghai | 57 | female | General Surgery | Blood | 0.06 |
|  | cgl170 | ST10 | Shanghai | 56 | male | General Surgery | Blood | 0.03 |
|  | cgl171 | ST7 | Shanghai | 54 | female | General Surgery | Blood | 0.12 |
|  | cgl172 | ST7 | Shanxi | 54 | female | ICU | Blood | 0.06 |
|  | cgl173 | ST7 | Shanxi | 58 | female | Pulmonology | Blood | 0.06 |
|  | cgl174 | ST7 | Shanxi | 76 | male | Pulmonology | Blood | 0.06 |
|  | cgl175 | ST10 | Liaoning | 88 | female | ICU | Blood | 0.03 |
|  | cgl177 | ST3 | Liaoning | 47 | male | ICU | Blood | 0.06 |

Table S2. Specimen type and sequence type analysis of sample sources

| Type | OR | Chi_square | P_value |
| --- | --- | --- | --- |
| Secretion | 9.67 | 3.08 | 0.0793 |
| Lavage Fluid | 4.79 | 1.72 | 0.1895 |
| Blood | 1.5 | 0.77 | 0.3797 |
| Tissue | Inf | 0.34 | 0.5624 |
| Catheter | 2.05 | 0.05 | 0.8233 |
| Drainage Fluid | 1.37 | 0.03 | 0.8672 |
| Ascite | 1.25 | 0 | 1 |
| Pus | 1.51 | 0 | 1 |
| Puncture Fluid | Inf | 0 | 1 |
| Bile | Inf | 0 | 1 |
| Pleural Effusion | Inf | 0 | 1 |
| Replacement Fluid | Inf | 0 | 1 |

**Table S3. Echinocandin MICs of 39 Caspofungin Heteroresistant Strains**

| STRAINS | ANF | MCF | CAS | FC | PZ | VOR | IZ | FZ | AB |
| --- | --- | --- | --- | --- | --- | --- | --- | --- | --- |
| cgl7 | 0.06 | 0.015 | 0.03 | ≤0.06 | 1 | 0.25 | 0.5 | 8 | 0.25 |
| cgl9 | 0.06 | 0.015 | 0.06 | ≤0.06 | 4 | 2 | 2 | 64 | 0.25 |
| cgl10 | 0.06 | 0.015 | 0.03 | ≤0.06 | 1 | 0.25 | 0.5 | 4 | 0.25 |
| cgl12 | 0.03 | 0.015 | 0.06 | ≤0.06 | 1 | 0.25 | 0.5 | 4 | 0.25 |
| cgl18 | 0.06 | 0.015 | 0.12 | ≤0.06 | 1 | 0.25 | 0.5 | 8 | 0.5 |
| cgl19 | 0.03 | 0.015 | 0.06 | ≤0.06 | 0.5 | 0.25 | 0.5 | 4 | 0.25 |
| cgl26 | 0.06 | 0.015 | 0.03 | ≤0.06 | 1 | 0.25 | 0.5 | 8 | 0.25 |
| cgl27 | 0.06 | 0.015 | 0.06 | ≤0.06 | 1 | 0.25 | 0.5 | 8 | 0.25 |
| cgl28 | 0.12 | 0.03 | 0.12 | ≤0.06 | 1 | 0.5 | 0.5 | 8 | 0.25 |
| cgl34 | 0.03 | 0.015 | 0.03 | ≤0.06 | 1 | 0.12 | 0.5 | 4 | ≤0.12 |
| cgl36 | 0.06 | 0.015 | 0.06 | ≤0.06 | 1 | 0.5 | 0.5 | 16 | 0.25 |
| cgl41 | 0.12 | 0.015 | 0.06 | ≤0.06 | 0.5 | 0.25 | 0.25 | 4 | 0.5 |
| cgl43 | 0.12 | 0.015 | 0.12 | ≤0.06 | >8 | 4 | >16 | 128 | 0.25 |
| cgl44 | 0.06 | ≤0.008 | 0.06 | ≤0.06 | 0.25 | 0.12 | 0.25 | 2 | 0.125 |
| cgl45 | 0.03 | 0.015 | 0.06 | ≤0.06 | 0.5 | 0.25 | 0.5 | 4 | 0.25 |
| cgl52 | 0.03 | ≤0.008 | 0.06 | ≤0.06 | 0.25 | 0.12 | 0.12 | 4 | 0.25 |
| cgl55 | 0.03 | ≤0.008 | 0.03 | ≤0.06 | 0.5 | 0.25 | 0.25 | 4 | ≤0.12 |
| cgl59 | 0.03 | 0.015 | 0.06 | ≤0.06 | 0.5 | 0.25 | 0.25 | 4 | 0.25 |
| cgl61 | 0.06 | 0.015 | 0.06 | ≤0.06 | 2 | 1 | 1 | 64 | 0.5 |
| cgl67 | 0.12 | 0.03 | 0.12 | ≤0.06 | 0.5 | 0.25 | 0.5 | 8 | 0.25 |
| cgl69 | 0.06 | 0.015 | 0.06 | ≤0.06 | 1 | 0.5 | 0.5 | 8 | 0.5 |
| cgl74 | 0.06 | 0.015 | 0.06 | ≤0.06 | 1 | 0.25 | 0.5 | 8 | 0.5 |
| cgl84 | 0.06 | 0.015 | 0.03 | ≤0.06 | 0.25 | 0.06 | 0.12 | 2 | ≤0.12 |
| cgl94 | 0.03 | 0.015 | 0.06 | ≤0.06 | 0.5 | 0.25 | 0.25 | 4 | 0.5 |
| cgl99 | 0.03 | 0.015 | 0.06 | ≤0.06 | 0.5 | 0.25 | 0.5 | 4 | 0.25 |
| cgl100 | 0.03 | 0.015 | 0.03 | ≤0.06 | 1 | 0.25 | 0.5 | 8 | 0.5 |
| cgl102 | 0.12 | 0.015 | 0.12 | ≤0.06 | 0.5 | 0.12 | 0.5 | 4 | 0.25 |
| cgl103 | 0.06 | 0.015 | 0.03 | ≤0.06 | 0.5 | 0.12 | 0.25 | 4 | 0.25 |
| cgl106 | 0.06 | 0.015 | 0.12 | ≤0.06 | 0.5 | 0.12 | 0.25 | 4 | 0.25 |
| cgl108 | 0.06 | 0.015 | 0.03 | ≤0.06 | 0.5 | 0.12 | 0.25 | 4 | 0.25 |
| cgl113 | 0.06 | 0.015 | 0.03 | ≤0.06 | 1 | 0.5 | 0.5 | 16 | 0.5 |
| cgl121 | 0.12 | 0.015 | 0.12 | ≤0.06 | 0.5 | 0.25 | 0.5 | 8 | 0.25 |
| cgl132 | 0.12 | 0.015 | 0.12 | ≤0.06 | 1 | 0.5 | 1 | 16 | 0.5 |
| cgl138 | 0.12 | 0.015 | 0.12 | ≤0.06 | 1 | 0.25 | 0.5 | 8 | 0.25 |
| cgl152 | 0.06 | 0.015 | 0.12 | ≤0.06 | 2 | 1 | 1 | 32 | 0.5 |
| cgl160 | 0.12 | 0.015 | 0.12 | ≤0.06 | 1 | 0.25 | 0.5 | 8 | 0.5 |
| cgl164 | 0.03 | 0.015 | 0.06 | ≤0.06 | 0.5 | 0.12 | 0.25 | 4 | 0.25 |
| cgl176 | 0.06 | ≤0.008 | 0.12 | ≤0.06 | 1 | 0.5 | 0.5 | 16 | 0.25 |
| cgl180 | 0.12 | ≤0.008 | 0.12 | ≤0.06 | 0.5 | 0.12 | 0.25 | 4 | 0.25 |

**Table S4. Caspofungin MICs Under both 30°C and 37°C**

| Strains | 30°C | 37°C |
| --- | --- | --- |
| CBS138 | 0.06 | 0.06 |
| cgl19 | 0.06 | 0.06 |
| cgl26 | 0.03 | 0.03 |
| cgl34 | 0.03 | 0.03 |
| cgl45 | 0.06 | 0.06 |
| cgl52 | 0.06 | 0.06 |
| cgl84 | 0.03 | 0.03 |
| cgl121 | 0.12 | 0.12 |
| cgl138 | 0.12 | 0.12 |
| cgl176 | 0.12 | 0.12 |

**Table S5. Core Candidates with High Module Membership and Gene Significance**

| name | Module Membership | Gene Significance | kWithin |
| --- | --- | --- | --- |
| *CAGL0A00231g* | 0.836957326 | 0.677950213 | 3.487887147 |
| *CAGL0A01221g* | 0.9474603 | 0.720737856 | 14.49200301 |
| *CAGL0A01606g* | 0.807922074 | 0.625959017 | 2.413159903 |
| *CAGL0A03608g* | 0.8419265 | 0.627342079 | 4.353889149 |
| *CAGL0A03828g* | 0.90733578 | 0.704165929 | 9.340987915 |
| *CAGL0A04081g* | 0.898291611 | 0.767217107 | 11.18097345 |
| *CAGL0A04169g* | 0.892000822 | 0.743784991 | 5.858902801 |
| *CAGL0A04829g* | 0.906746689 | 0.790266342 | 11.4656916 |
| *CAGL0B00990g* | 0.891538384 | 0.694416676 | 6.886121429 |
| *CAGL0B01188g* | 0.94086068 | 0.775357703 | 14.83078268 |
| *CAGL0B02145g* | 0.881530714 | 0.633843566 | 6.061925299 |
| *CAGL0B03707g* | 0.839820325 | 0.639058287 | 3.948064414 |
| *CAGL0B03839g* | 0.925665857 | 0.686568433 | 11.04942056 |
| *CAGL0B04389g* | 0.924631398 | 0.722433389 | 9.947414068 |
| *CAGL0B04873g* | 0.901504053 | 0.699206824 | 9.293049299 |
| *CAGL0C02211g* | 0.899548373 | 0.664893988 | 8.370434762 |
| *CAGL0C02321g* | 0.888439407 | 0.801271044 | 6.130153173 |
| *CAGL0C02431g* | 0.91158211 | 0.738374733 | 7.619101492 |
| *CAGL0C03487g* | 0.856739938 | 0.693796235 | 4.839467519 |
| *CAGL0C04114g* | 0.840611262 | 0.680133795 | 4.447968411 |
| *CAGL0C04136g* | 0.851801781 | 0.780272458 | 7.306265668 |
| *CAGL0C04389g* | 0.842415278 | 0.721253099 | 5.87968886 |
| *CAGL0C04411g* | 0.902825449 | 0.76170487 | 11.17741794 |
| *CAGL0D02090g* | 0.861379821 | 0.79025535 | 7.481668417 |
| *CAGL0D02530g* | 0.876763364 | 0.773308982 | 7.365233696 |
| *CAGL0D02640g* | 0.876057294 | 0.64663422 | 8.961106258 |
| *CAGL0D02662g* | 0.879262913 | 0.655939365 | 9.309685053 |
| *CAGL0D04620g* | 0.908268601 | 0.784202513 | 10.15200459 |
| *CAGL0D04642g* | 0.886970117 | 0.750590524 | 4.520463878 |
| *CAGL0D04752g* | 0.966097353 | 0.782026097 | 19.12509837 |
| *CAGL0D06402g* | 0.940133249 | 0.68278268 | 12.55493048 |
| *CAGL0E01331g* | 0.940808174 | 0.730849426 | 15.11034389 |
| *CAGL0E02123g* | 0.905771684 | 0.654096291 | 7.468112834 |
| *CAGL0E02145g* | 0.916933434 | 0.737096744 | 12.06623358 |
| *CAGL0E02255g* | 0.853031984 | 0.803435881 | 3.161657917 |
| *CAGL0E02409g* | 0.87651052 | 0.654021338 | 7.731502926 |
| *CAGL0E04180g* | 0.900550194 | 0.702813098 | 9.253905821 |
| *CAGL0E04620g* | 0.857288408 | 0.624268735 | 4.791479768 |
| *CAGL0E04774g* | 0.888992188 | 0.804862653 | 5.590001186 |
| *CAGL0E06028g* | 0.854131756 | 0.633858939 | 2.929575775 |
| *CAGL0F01287g* | 0.92120819 | 0.754635108 | 10.71835884 |
| *CAGL0F01485g* | 0.859061045 | 0.633860727 | 4.015295024 |
| *CAGL0F02079g* | 0.829058146 | 0.668484819 | 6.313512276 |
| *CAGL0F03773g* | 0.830974575 | 0.750739848 | 4.61859864 |
| *CAGL0F04763g* | 0.903485294 | 0.70446995 | 8.444118779 |
| *CAGL0F05329g* | 0.847216116 | 0.721812691 | 5.143750198 |
| *CAGL0F05885g* | 0.883339688 | 0.700624846 | 7.711856395 |
| *CAGL0F07337g* | 0.875459952 | 0.638396605 | 5.199265672 |
| *CAGL0F07579g* | 0.864992424 | 0.741561223 | 7.011508554 |
| *CAGL0F07601g* | 0.881684477 | 0.733452594 | 7.60315611 |
| *CAGL0F08107g* | 0.932336565 | 0.779743994 | 10.65085495 |
| *CAGL0F08833g* | 0.858209481 | 0.697480655 | 5.455611449 |
| *CAGL0G00286g* | 0.856358201 | 0.731925829 | 5.525696271 |
| *CAGL0G00858g* | 0.884633068 | 0.776124647 | 5.732580749 |
| *CAGL0G01408g* | 0.915188189 | 0.679261052 | 9.630753191 |
| *CAGL0G02123g* | 0.946651317 | 0.800159814 | 11.41955338 |
| *CAGL0G02387g* | 0.943176186 | 0.714870294 | 13.39245529 |
| *CAGL0G02585g* | 0.919241189 | 0.71236171 | 11.59751794 |
| *CAGL0G02937g* | 0.875446017 | 0.802535647 | 4.571088484 |
| *CAGL0G04499g* | 0.915466048 | 0.739949033 | 11.35183035 |
| *CAGL0G04851g* | 0.910687338 | 0.783067272 | 13.66912668 |
| *CAGL0G05269g* | -0.861939668 | -0.655348904 | 4.747990625 |
| *CAGL0G05698g* | 0.91353059 | 0.740870535 | 11.77792363 |
| *CAGL0G06666g* | 0.822949624 | 0.633042402 | 4.797818344 |
| *CAGL0G07249g* | 0.875913343 | 0.631205125 | 6.257601355 |
| *CAGL0G07403g* | 0.827082224 | 0.66209367 | 2.682667288 |
| *CAGL0G08668g* | 0.92268769 | 0.67403905 | 10.79948631 |
| *CAGL0G08954g* | 0.920154122 | 0.732648273 | 12.15253356 |
| *CAGL0G09449g* | 0.883353335 | 0.764390207 | 9.087889918 |
| *CAGL0G09515g* | 0.930696254 | 0.792795899 | 14.30225381 |
| *CAGL0H00594g* | 0.859317452 | 0.631438223 | 3.292704373 |
| *CAGL0H00638g* | 0.853473482 | 0.712793379 | 5.414017443 |
| *CAGL0H01331g* | 0.900539894 | 0.75050066 | 5.322868404 |
| *CAGL0H02255g* | 0.920966665 | 0.773422313 | 13.79057156 |
| *CAGL0H02695g* | 0.889537906 | 0.806671141 | 8.338173939 |
| *CAGL0H03619g* | 0.935503181 | 0.744699408 | 13.74689848 |
| *CAGL0H07557g* | -0.812239637 | -0.735295016 | 3.371453232 |
| *CAGL0H07997g* | 0.856815252 | 0.726740971 | 3.816384311 |
| *CAGL0H08541g* | 0.927117452 | 0.766947086 | 11.32240376 |
| *CAGL0H08624g* | 0.882056606 | 0.660390274 | 4.846124392 |
| *CAGL0H09834g* | 0.881556894 | 0.775073511 | 9.183979218 |
| *CAGL0H09856g* | 0.890382653 | 0.751348605 | 9.389366217 |
| *CAGL0I00286g* | 0.837684659 | 0.675738432 | 4.864424912 |
| *CAGL0I00484g* | 0.956097538 | 0.767458271 | 16.53613274 |
| *CAGL0I00550g* | 0.808845749 | 0.710565978 | 2.383571111 |
| *CAGL0I01210g* | 0.815373535 | 0.611733043 | 2.764364758 |
| *CAGL0I01914g* | 0.92943501 | 0.798825 | 12.71926423 |
| *CAGL0I02178g* | 0.944137349 | 0.740291785 | 15.57755024 |
| *CAGL0I04796g* | 0.814679849 | 0.723143906 | 2.125230973 |
| *CAGL0I04818g* | 0.893363901 | 0.717775147 | 10.76749113 |
| *CAGL0I04994g* | 0.948807253 | 0.748389625 | 14.83722576 |
| *CAGL0I05764g* | 0.88125771 | 0.740437942 | 5.938720137 |
| *CAGL0I05852g* | 0.901925504 | 0.66945161 | 9.568057117 |
| *CAGL0I06160g* | 0.892918797 | 0.7721423 | 10.62525506 |
| *CAGL0I06182g* | 0.824309859 | 0.744635029 | 6.922583342 |
| *CAGL0I06204g* | 0.822823903 | 0.7556871 | 6.308092046 |
| *CAGL0I06644g* | 0.964057673 | 0.742851427 | 15.72072374 |
| *CAGL0I07425g* | 0.810326875 | 0.670696839 | 1.551458684 |
| *CAGL0I08591g* | -0.848883794 | -0.780298747 | 3.27443039 |
| *CAGL0I08723g* | 0.860547759 | 0.616353556 | 2.967283832 |
| *CAGL0I08745g* | 0.928478605 | 0.735772099 | 10.00436825 |
| *CAGL0I09064g* | 0.859923745 | 0.732075562 | 6.308584555 |
| *CAGL0I09746g* | 0.913083343 | 0.755527609 | 11.15396567 |
| *CAGL0I10901g* | 0.842870891 | 0.654454479 | 6.63040222 |
| *CAGL0J02750g* | 0.851648827 | 0.686764714 | 3.953880486 |
| *CAGL0J02970g* | 0.873652194 | 0.691771315 | 5.826461264 |
| *CAGL0J03124g* | 0.843880585 | 0.702721671 | 6.335483327 |
| *CAGL0J03212g* | 0.909796692 | 0.66369374 | 10.28173978 |
| *CAGL0J04466g* | 0.805517352 | 0.708933392 | 3.464140801 |
| *CAGL0J04950g* | 0.884269174 | 0.704208966 | 7.965043976 |
| *CAGL0J06798g* | 0.895111505 | 0.729192474 | 8.667458694 |
| *CAGL0J08437g* | 0.892697081 | 0.682755213 | 8.251538095 |
| *CAGL0J08822g* | 0.912370668 | 0.739060553 | 9.148854591 |
| *CAGL0J09240g* | 0.898384384 | 0.743633623 | 9.93931981 |
| *CAGL0J09328g* | 0.90183159 | 0.76376487 | 11.6868319 |
| *CAGL0J10076g* | 0.932748485 | 0.708578848 | 12.71423351 |
| *CAGL0J10208g* | 0.862978239 | 0.6065809 | 6.034277269 |
| *CAGL0J10318g* | 0.921292629 | 0.756895599 | 12.88389184 |
| *CAGL0J11132g* | 0.910062747 | 0.717281963 | 10.85675188 |
| *CAGL0J11506g* | 0.837560106 | 0.734878855 | 4.130937013 |
| *CAGL0J11638g* | 0.951881808 | 0.785929898 | 15.35560343 |
| *CAGL0J11748g* | 0.881949579 | 0.718033842 | 9.667537234 |
| *CAGL0K00715g* | 0.958327971 | 0.781193873 | 15.34012248 |
| *CAGL0K00737g* | 0.832872219 | 0.736742665 | 5.405560627 |
| *CAGL0K00913g* | 0.84153841 | 0.717691975 | 7.684329341 |
| *CAGL0K04367g* | 0.861598976 | 0.777784893 | 5.630685348 |
| *CAGL0K05445g* | 0.927083632 | 0.774738691 | 14.60552764 |
| *CAGL0K08272g* | 0.834256922 | 0.786400645 | 3.248666353 |
| *CAGL0K09130g* | 0.91253932 | 0.761091685 | 13.21332545 |
| *CAGL0K10626g* | 0.917101892 | 0.740987052 | 14.75304224 |
| *CAGL0K11440g* | 0.899233279 | 0.765041962 | 9.93767355 |
| *CAGL0K11462g* | 0.877692888 | 0.74230175 | 6.706406649 |
| *CAGL0K11506g* | 0.854498605 | 0.632789852 | 7.95549432 |
| *CAGL0K11946g* | 0.916396865 | 0.772485011 | 7.700160925 |
| *CAGL0K12848g* | 0.87059431 | 0.699997453 | 9.506457822 |
| *CAGL0L01749g* | 0.92128079 | 0.747843427 | 8.350544776 |
| *CAGL0L03696g* | 0.853788659 | 0.623231905 | 3.698805908 |
| *CAGL0L04026g* | -0.870027113 | -0.696957754 | 6.330188453 |
| *CAGL0L04136g* | 0.871229998 | 0.664207148 | 4.029279177 |
| *CAGL0L06072g* | 0.88900789 | 0.797276885 | 5.915595657 |
| *CAGL0L06424g* | 0.882660597 | 0.787155941 | 8.481682251 |
| *CAGL0L06512g* | 0.8426486 | 0.641983139 | 4.926688699 |
| *CAGL0L06996g* | 0.872340011 | 0.637385438 | 4.956311351 |
| *CAGL0L07480g* | 0.844949382 | 0.688021864 | 4.702541981 |
| *CAGL0L07502g* | 0.882288284 | 0.801156825 | 9.187334679 |
| *CAGL0L08547g* | 0.959047313 | 0.8026131 | 14.34895531 |
| *CAGL0L09581g* | 0.886404717 | 0.648121663 | 6.626440586 |
| *CAGL0L10252g* | 0.906845238 | 0.716208147 | 7.429744728 |
| *CAGL0L10648g* | 0.872837875 | 0.680579629 | 5.370023502 |
| *CAGL0L10714g* | 0.920996975 | 0.713004528 | 13.23650192 |
| *CAGL0L11132g* | 0.962647493 | 0.797482065 | 16.12910773 |
| *CAGL0L13222g* | 0.900943874 | 0.702518564 | 9.070554349 |
| *CAGL0M02189g* | 0.906257508 | 0.752360604 | 7.915395205 |
| *CAGL0M02783g* | 0.904361266 | 0.77862591 | 8.591457322 |
| *CAGL0M03179g* | 0.819934732 | 0.724333151 | 3.779005346 |
| *CAGL0M03773g* | 0.943398284 | 0.728214344 | 14.1118524 |
| *CAGL0M04499g* | 0.96360497 | 0.831307993 | 15.788471 |
| *CAGL0M05599g* | 0.821515263 | 0.725369108 | 3.937696568 |
| *CAGL0M05621g* | 0.923494154 | 0.697074684 | 8.209217087 |
| *CAGL0M05841g* | 0.929415282 | 0.769680731 | 10.33028143 |
| *CAGL0M06435g* | 0.833208401 | 0.630718271 | 3.515166205 |
| *CAGL0M08514g* | 0.887696615 | 0.800957292 | 11.45543772 |
| *CAGL0M08756g* | 0.889929848 | 0.679043659 | 5.992324701 |
| *CAGL0M09086g* | 0.910408458 | 0.677324063 | 9.685298885 |
| *CAGL0M11682g* | -0.830737711 | -0.730214088 | 2.897409969 |
| *CAGL0M12254g* | 0.801812971 | 0.620545966 | 2.026702055 |
| *CAGL0M12793g* | -0.859381612 | -0.765245761 | 3.019331663 |
| *CAGL0M12947g* | -0.858964671 | -0.699182495 | 9.014031955 |
| *CAGL0M13805g* | 0.911265892 | 0.749754634 | 11.69044896 |

**Table S6. Parental and Descendant Strain Caspofungin MICs**

| STRAIN | Parental MIC | Descendant MIC | Descendant After Drug-Free Passage | *FKS2* Mutations |
| --- | --- | --- | --- | --- |
| cgl9-1 | 0.06 | 0.03 | / | *F659S* |
| cgl9-2 | 0.06 | 0.12 | / | *F659C* |
| cgl10-1 | 0.03 | 0.12 | / | *S663F* |
| cgl12-1 | 0.06 | 8 | 8 | *F659del* |
| cgl12-2 | 0.06 | 0.25 | / | */* |
| cgl18-2 | 0.12 | 0.5 | 0.5 | */* |
| cgl19-1 | 0.06 | 1 | 1 | */* |
| cgl26-1 | 0.03 | 0.25 | / | *F659del* |
| cgl26-2 | 0.03 | 0.5 | 0.5 | */* |
| cgl27-1 | 0.06 | 0.5 | 0.5 | *F659del* |
| cgl27-2 | 0.06 | 0.25 | / | */* |
| cgl28-1 | 0.12 | 0.5 | 0.5 | */* |
| cgl34-1 | 0.03 | 1 | 1 | */* |
| cgl36-1 | 0.06 | 1 | 1 | */* |
| cgl41-1 | 0.06 | 0.25 | / | */* |
| cgl43-1 | 0.12 | 0.25 | / | */* |
| cgl43-2 | 0.12 | 0.06 | / | */* |
| cgl44-1 | 0.06 | 8 | 8 | *F659del* |
| cgl44-2 | 0.06 | 0.25 | / | */* |
| cgl45-1 | 0.06 | 1 | 1 | *F659del* |
| cgl45-2 | 0.06 | 0.25 | / | */* |
| cgl52-1 | 0.06 | 0.015 | / | */* |
| cgl52-2 | 0.06 | 0.015 | / | */* |
| cgl55-1 | 0.03 | 0.25 | / | */* |
| cgl55-2 | 0.03 | 0.25 | / | */* |
| cgl59-1 | 0.06 | 8 | 8 | *F659del* |
| cgl59-2 | 0.06 | 2 | 2 | *F659del* |
| cgl61-1 | 0.06 | 0.25 | / | */* |
| cgl67-1 | 0.12 | 0.5 | 0.5 | */* |
| cgl67-2 | 0.12 | 0.5 | 0.5 | */* |
| cgl69-1 | 0.06 | 0.25 | / | */* |
| cgl69-2 | 0.06 | 0.12 | / | */* |
| cgl74-1 | 0.06 | 0.25 | / | */* |
| cgl84-2 | 0.03 | 0.06 | / | */* |
| cgl94-1 | 0.06 | 0.25 | / | */* |
| cgl94-2 | 0.06 | 0.25 | / | *P667L* |
| cgl99-1 | 0.06 | 0.5 | 0.5 | */* |
| cgl100-1 | 0.03 | 0.5 | 0.5 | */* |
| cgl100-2 | 0.03 | 0.5 | 0.5 | */* |
| cgl102-1 | 0.12 | 0.03 | / | */* |
| cgl102-2 | 0.12 | 0.25 | / | */* |
| cgl106-1 | 0.12 | 0.25 | / | */* |
| cgl106-2 | 0.12 | 0.06 | / | */* |
| cgl113-1 | 0.06 | 0.12 | / | */* |
| cgl113-2 | 0.06 | 0.12 | / | */* |
| cgl121-1 | 0.12 | 2 | 2 | */* |
| cgl121-2 | 0.12 | 1 | 1 | *F659del* |
| cgl138-1 | 0.12 | 0.12 | / | */* |
| cgl138-2 | 0.12 | 0.5 | 0.5 | */* |
| cgl152-1 | 0.12 | 4 | 4 | *F659del* |
| cgl152-2 | 0.12 | 0.25 | / | */* |
| cgl160-1 | 0.12 | 0.5 | 0.5 | *R1378S* |
| cgl160-2 | 0.12 | 0.25 | / | */* |
| cgl164-1 | 0.06 | 0.25 | / | */* |
| cgl164-2 | 0.06 | 0.25 | / | */* |
| cgl176-1 | 0.12 | 0.5 | 0.5 | */* |
| cgl176-2 | 0.12 | 0.5 | 0.5 | */* |
| cgl180-1 | 0.12 | 0.25 | / | *L664R* |
| cgl180-2 | 0.12 | 0.25 | / | *L660M* |

**Table S7. Copy Number Variations Analysis of Resistant Subclones (Window 5kb)**

| Sample Name | Chromosome | Start Position | End Position | Variant Classification | Copy Number |
| --- | --- | --- | --- | --- | --- |
| cgl102-1 | ChrL | 1425000 | 1440000 | Deletion | 0 |
| cgl102-2 | ChrL | 1420000 | 1440000 | Deletion | 0 |
| cgl138-1 | ChrJ | 0 | 485000 | Amplification | 2 |
| cgl138-1 | ChrJ | 490000 | 1155000 | Amplification | 2 |
| cgl138-1 | ChrK | 1240000 | 1255000 | Deletion | 0 |
| cgl138-2 | ChrF | 0 | 885000 | Amplification | 2 |
| cgl152-2 | ChrA | 20000 | 460000 | Amplification | 2 |
| cgl152-2 | ChrB | 25000 | 80000 | Amplification | 2 |
| cgl152-2 | ChrB | 320000 | 430000 | Amplification | 2 |
| cgl176-2 | ChrE | 0 | 30000 | Amplification | 2 |
| cgl26-2 | ChrJ | 0 | 1170000 | Amplification | 2 |
| cgl36-1 | ChrG | 10000 | 925000 | Amplification | 2 |
| cgl43-1 | ChrB | 0 | 50000 | Amplification | 2 |
| cgl67-1 | ChrA | 270000 | 280000 | Amplification | 2 |
| cgl67-1 | ChrE | 95000 | 110000 | Amplification | 2 |
| cgl67-1 | ChrH | 1030000 | 1035000 | Deletion | 0 |
| cgl67-1 | ChrM | 760000 | 770000 | Amplification | 2 |
| cgl67-2 | ChrA | 260000 | 270000 | Amplification | 2 |
| cgl67-2 | ChrE | 95000 | 110000 | Amplification | 2 |
| cgl67-2 | ChrL | 1420000 | 1440000 | Deletion | 0 |
| cgl67-2 | ChrM | 760000 | 770000 | Amplification | 2 |
| cgl84-1 | ChrE | 0 | 10000 | Deletion | 0 |
| cgl84-1 | ChrG | 665000 | 670000 | Amplification | 2 |
| cgl84-1 | ChrG | 670000 | 675000 | Amplification | 5 |
| cgl84-1 | ChrH | 1010000 | 1025000 | Deletion | 0 |
| cgl84-1 | ChrI | 970000 | 975000 | Deletion | 0 |
| cgl84-1 | ChrJ | 480000 | 485000 | Deletion | 0 |
| cgl84-1 | ChrK | 1165000 | 1175000 | Deletion | 0 |
| cgl94-2 | ChrH | 1030000 | 1035000 | Deletion | 0 |
| cgl99-1 | ChrD | 110000 | 115000 | Amplification | 2 |
| cgl99-1 | ChrE | 535000 | 555000 | Amplification | 2 |
| cgl99-1 | ChrJ | 495000 | 510000 | Amplification | 2 |
| cgl99-1 | ChrM | 1035000 | 1060000 | Amplification | 2 |
| cgl12-2 | ChrA | 15000 | 405000 | Amplification | 2 |
| cgl12-2 | ChrJ | 0 | 35000 | Amplification | 2 |
| cgl12-2 | ChrJ | 35000 | 285000 | Amplification | 3 |
| cgl12-2 | ChrJ | 285000 | 940000 | Amplification | 2 |
| cgl12-2 | ChrJ | 940000 | 1155000 | Amplification | 3 |
| cgl160-2 | ChrF | 0 | 920000 | Amplification | 2 |

**Table S8. Copy Number Variations Analysis of Resistant Subclones (Window 1kb, only ChrJ)**

| Sample Name | Start Position | End Position | Variant Classification | Copy Number |
| --- | --- | --- | --- | --- |
| cgl100-2 | 487000 | 489000 | Deletion | 0 |
| cgl102-2 | 164000 | 169000 | Deletion | 0 |
| cgl138-1 | 0 | 163000 | Amplification | 2 |
| cgl138-1 | 163000 | 165000 | Deletion | 0 |
| cgl138-1 | 167000 | 486000 | Amplification | 2 |
| cgl138-1 | 488000 | 1159000 | Amplification | 2 |
| cgl138-2 | 163000 | 165000 | Deletion | 0 |
| cgl138-2 | 486000 | 488000 | Deletion | 0 |
| cgl161-1 | 0 | 165000 | Amplification | 2 |
| cgl176-1 | 167000 | 169000 | Deletion | 0 |
| cgl180-1 | 164000 | 169000 | Deletion | 0 |
| cgl180-2 | 167000 | 171000 | Deletion | 0 |
| cgl180-2 | 490000 | 491000 | Deletion | 0 |
| cgl18-1 | 485000 | 487000 | Deletion | 0 |
| cgl26-1 | 164000 | 165000 | Deletion | 0 |
| cgl26-2 | 2000 | 164000 | Amplification | 2 |
| cgl26-2 | 166000 | 167000 | Deletion | 0 |
| cgl26-2 | 167000 | 488000 | Amplification | 2 |
| cgl26-2 | 491000 | 1173000 | Amplification | 2 |
| cgl27-1 | 487000 | 489000 | Deletion | 0 |
| cgl27-2 | 252000 | 253000 | Amplification | 2 |
| cgl28-1 | 166000 | 167000 | Deletion | 0 |
| cgl34-1 | 200000 | 201000 | Amplification | 2 |
| cgl36-1 | 164000 | 165000 | Deletion | 0 |
| cgl36-2 | 166000 | 171000 | Deletion | 0 |
| cgl41-1 | 165000 | 167000 | Deletion | 0 |
| cgl41-1 | 168000 | 169000 | Deletion | 0 |
| cgl41-1 | 489000 | 490000 | Deletion | 0 |
| cgl41-2 | 165000 | 167000 | Deletion | 0 |
| cgl41-2 | 488000 | 490000 | Deletion | 0 |
| cgl43-2 | 488000 | 490000 | Deletion | 0 |
| cgl45-1 | 152000 | 154000 | Deletion | 0 |
| cgl45-2 | 152000 | 154000 | Deletion | 0 |
| cgl45-2 | 492000 | 493000 | Amplification | 2 |
| cgl52-2 | 487000 | 489000 | Deletion | 0 |
| cgl59-2 | 163000 | 168000 | Deletion | 0 |
| cgl67-1 | 162000 | 167000 | Deletion | 0 |
| cgl67-2 | 486000 | 489000 | Deletion | 0 |
| cgl69-1 | 161000 | 165000 | Deletion | 0 |
| cgl69-2 | 163000 | 165000 | Deletion | 0 |
| cgl74-1 | 162000 | 167000 | Deletion | 0 |
| cgl74-1 | 487000 | 488000 | Deletion | 0 |
| cgl84-1 | 159000 | 160000 | Amplification | 2 |
| cgl84-1 | 480000 | 482000 | Deletion | 0 |
| cgl84-2 | 164000 | 166000 | Deletion | 0 |
| cgl9-2 | 164000 | 166000 | Deletion | 0 |
| cgl94-1 | 163000 | 168000 | Deletion | 0 |
| cgl94-2 | 163000 | 166000 | Deletion | 0 |
| cgl94-2 | 487000 | 489000 | Deletion | 0 |
| cgl99-1 | 193000 | 229000 | Amplification | 2 |
| cgl99-1 | 494000 | 509000 | Amplification | 2 |
| cgl12-2 | 0 | 37000 | Amplification | 2 |
| cgl12-2 | 37000 | 164000 | Amplification | 3 |
| cgl12-2 | 166000 | 296000 | Amplification | 3 |
| cgl12-2 | 296000 | 870000 | Amplification | 2 |
| cgl12-2 | 870000 | 1155000 | Amplification | 3 |
| cgl44-2 | 77000 | 164000 | Amplification | 2 |

**Table S9. Top 10 Features Screened by Multi-Generative Model**

| Feature | Correlation | Correlation Strength | adjusted p value | effect size | Effect Magnitude |
| --- | --- | --- | --- | --- | --- |
| *FKS2_F659del* | 0.574 | Strong | 0.00094 | 3.316 | Very Large |
| *PIR2_G149_I167del* | 0.542 | Strong | 0.00143 | 2.485 | Very Large |
| *CAGL0A02233g_N470S* | 0.421 | Moderate | 0.02933 | 1.875 | Large |
| *CAGL0K07502g_Y105C* | 0.409 | Moderate | 0.02933 | 1.65 | Large |
| *CAGL0A02299g_G197fs* | 0.371 | Moderate | 0.05229 | 1.581 | Large |
| *CAGL0A02233g_N470D* | 0.362 | Moderate | 0.05229 | 1.462 | Large |
| *CAGL0A02299g_A198fs* | 0.272 | Weak | 0.21135 | 1.081 | Large |
| *CAGL0L09251g_E153_E157del* | 0.217 | Weak | 0.37133 | 1.5 | Large |
| *CAGL0A02299g_V204fs* | 0.206 | Weak | 0.37133 | 0.818 | Medium |
| *CAGL0A02299g_G201fs* | 0.203 | Weak | 0.37133 | 0.807 | Medium |

**Table S10. Primer Sequences**

| **Gene** | **Primer** | **Sequences** |
| --- | --- | --- |
| *CRZ1* | *CRZ1-upF* | GGAAATGATGTGGTGGACATGTAC |
|  | *CRZ1-upR* | CGCCCATTGCTGAATATTGC |
|  | *CRZ1-NATf* | GCAATATTCAGCAATGGGCGCGGCATCAGAGCAGATTGTA |
|  | *CRZ1-NATr* | CAAAGTAACACCCATCTCAGTTGCGCGTGAATGTAAGCGTGAC |
|  | *CRZ1-dwF* | GCAACTGAGATGGGTGTTACTTTG |
|  | *CRZ1-dwR* | CCAGCATCGAGGAGTTTTTTGC |
|  | *CRZ1-checkF* | CGGAGGCCCTTTAACATAGTACC |
|  | *CRZ1-checkR* | ATCGGTAAGCCGTGTCGTC |
|  | *Lys21-BackboneF* | CGGAGGCCCTTTAACATAGTACC |
|  | *Lys21-BackboneR* | GAATTCCTGCAGCCCGGG |
|  | *CRZ1 F for Lys21* | CCCCCGGGCTGCAGGAATTCGGAAATGATGTGGTGGACATGTAC |
|  | *CRZ1 R for Lys21* | TTCTCGAGGTCGACCTGCAGCTGCTTGCAACTGATCATCAG |
|  | *CRZ1-Lys21-checkF* | AAGACATGCATAAGGGCATCG |
|  | *CRZ1-Lys21-checkR* | CCAGAACCACAAGAAGCAGAAG |
| *CNB1* | *CNB1-upF* | GGACATCGTACATATCATGCAGTGC |
|  | *CNB1-upR* | TGCAGCTCCCATTTCCCTTG |
|  | *CNB1-NATf* | CAAGGGAAATGGGAGCTGCACGGCATCAGAGCAGATTGTA |
|  | *CNB1-NATr* | CCTCGTTAAGTTTCTTGCCTGAAGCGTGAATGTAAGCGTGAC |
|  | *CNB1-dwF* | TTCAGGCAAGAAACTTAACGAGG |
|  | *CNB1-dwR* | ATGAAAGGGCGATGATAACGC |
|  | *CNB1-checkF* | GTTGGATGGCTACACTCTTGAT |
|  | *CNB1-checkR* | ATCGGTAAGCCGTGTCGTC |
|  | *Lys21-BackboneF* | CGGAGGCCCTTTAACATAGTACC |
|  | *Lys21-BackboneR* | GAATTCCTGCAGCCCGGG |
|  | *CNB1 F for Lys21* | CCCCCGGGCTGCAGGAATTCTGCCAAACGTTACTTGCTGTGG |
|  | *CNB1 R for Lys21* | TTCTCGAGGTCGACCTGCAGATGAAAGGGCGATGATAACGC |
|  | *CNB1-Lys21-checkF* | GTGGTTGGGGACTGTCTCATC |
|  | *CNB1-Lys21-checkR* | CCAGAACCACAAGAAGCAGAAG |
| *PIR2* | *PIR2-upF* | CCCTCCCCGATCCTTTCTATC |
|  | *PIR2-upR* | GCACTTACAAGAACCAGCCC |
|  | *PIR2-NATF* | GGGCTGGTTCTTGTAAGTGCCGGCATCAGAGCAGATTGTA |
|  | *PIR2-NATR* | GAATGAAAGCCAGCCAGTGGGCGTGAATGTAAGCGTGAC |
|  | *PIR2-dwF* | CCACTGGCTGGCTTTCATTC |
|  | *PIR2-dwR* | GCCCATCTAGATCAACACACTC |
|  | *PIR2-delCheckF* | TCTTTGAATGGGGACGCCTCG |
|  | *PIR2-delCheckR* | ATCGGTAAGCCGTGTCGTC |
|  | *Lys21-BackboneF* | CTGCAGGTCGACCTCGAGAAC |
|  | *Lys21-BackboneF* | GAATTCCTGCAGCCCGGG |
|  | *PIR2-F for Lys21* | CCCCCGGGCTGCAGGAATTCGGGATCTGTCTCCTCGAGAG |
|  | *PIR2-R for Lys21* | TTCTCGAGGTCGACCTGCAGGGCTGGTCCATCACCGAAC |
|  | *PIR2-Lys21-CheckF* | GTCCGTAGAGAAGTGGGATC |
|  | *PIR2-Lys21-CheckR* | GGCCAAGGCGGAAGTAG |
| *FKS2* | *FKS2-upF* | TCTAAGTTACACCTTTGCCCCCTC |
|  | *FKS2-upR* | GCCGGGTTGGAATTTTCTTTTTCTG |
|  | *FKS2-NATF* | AAGAAAATTCCAACCCGGCCGGCATCAGAGCAGATTGTA |
|  | *FKS2-NATR* | TATCTATTGCCTCTGCGTGGCGTGAATGTAAGCGTGAC |
|  | *FKS2-dwF* | CACGCAGAGGCAATAGATAATCCC |
|  | *FKS2-dwR* | CTTGTCAGAACTGTTTTCGCCATC |
|  | *FKS2-delCheckF* | TTGCCTTCGTGATTTGTGTGG |
|  | *FKS2-delCheckR* | ATCGGTAAGCCGTGTCGTC |
